# On the role of diffusion dynamics on community-aware centrality measures

**DOI:** 10.1371/journal.pone.0306561

**Published:** 2024-07-18

**Authors:** Stephany Rajeh, Hocine Cherifi

**Affiliations:** 1 Efrei Research Lab, EFREI Paris-Pantheon-Assas University, Villejuif, France; 2 LIP6 CNRS, Sorbonne University, Paris, France; 3 ICB UMR 6303 CNRS, University of Burgundy, Dijon, France; Asansol Polytechnic, INDIA

## Abstract

Theoretical and empirical studies on diffusion models have revealed their versatile applicability across different fields, spanning from sociology and finance to biology and ecology. The presence of a community structure within real-world networks has a substantial impact on how diffusion processes unfold. Key nodes located both within and between these communities play a crucial role in initiating diffusion, and community-aware centrality measures effectively identify these nodes. While numerous diffusion models have been proposed in literature, very few studies investigate the relationship between the diffusive ability of key nodes selected by community-aware centrality measures, the distinct dynamical conditions of various models, and the diverse network topologies. By conducting a comparative evaluation across four diffusion models, utilizing both synthetic and real-world networks, along with employing two different community detection techniques, our study aims to gain deeper insights into the effectiveness and applicability of the community-aware centrality measures. Results suggest that the diffusive power of the selected nodes is affected by three main factors: the strength of the network’s community structure, the internal dynamics of each diffusion model, and the budget availability. Specifically, within the category of simple contagion models, such as SI, SIR, and IC, we observe similar diffusion patterns when the network’s community structure strength and budget remain constant. In contrast, the LT model, which falls under the category of complex contagion dynamics, exhibits divergent behavior under the same conditions.

## 1 Introduction

Diffusion processes in networks refer to the spread or dissemination of various elements within a networked system, such as information, behaviors, innovations, or diseases. These processes are pivotal in understanding how ideas, influences, or entities traverse through interconnected nodes or individuals. Whether it’s the transmission of information on social media, the propagation of a viral outbreak like the COVID-19 pandemic, or the rapid adoption of new technologies, such as smartphones and social media platforms, studying diffusion in networks provides insights into the dynamics of interconnected systems and their societal implications. Researchers introduce models and frameworks to understand better and manage these diffusion processes to mitigate negative consequences, such as pandemics, and maximize positive impacts, like efficient containment measures during health crises. Understanding diffusion processes in networks is crucial for learning the dynamics of interconnected systems and addressing various real-world challenges. Examining how information, behaviors, and innovations spread within networks allows us to devise strategies to tackle misinformation, epidemics, and optimized marketing campaigns. Moreover, this knowledge can empower us to harness the positive aspects of diffusion, such as spreading awareness, promoting positive messages, and fostering innovation. In essence, the study of diffusion processes in networks is at the intersection of social science, technology, and public health, offering valuable insights and opportunities for improving our interconnected world.

Diffusion begins from nodes located in specific areas of the network and spreads out with time. How to select the seed nodes to maximize diffusion is a fundamental problem. Centrality measures are one of the main approaches to do so. They rely on topological information from the network to quantify node importance. Since the community structure impacts the diffusion spreading dynamics [1–3], researchers showed that classical centrality measures may fallback in terms of selecting the most influential nodes [4–11]. Therefore, it is important to incorporate community structure information to select seed nodes that maximize diffusion.

Unlike the classical centrality measures, which focus more on either the local or the global influence of a node, the so-called community-aware centrality measures incorporate the node’s local and global influence through its intra-community and inter-community links, respectively [4–12]. The difference between these measures is how they combine these two types of links. If more importance is given to the intra-community links (i.e., local influence), the measure emphasizes hub-like nodes. Conversely, if importance is given to the inter-community links (i.e., global influence), the measure renders bridge-like nodes more important.

The diffusive ability of the community-aware centrality measures in selecting seed nodes is assessed in a dynamic spreading scenario with specific conditions set on nodes and/or edges. Most of the studies use the SIR model to assess the impact of the selection of seed nodes either to maximize diffusion or to minimize it (this can also be called immunization) [4–10]. Despite being widely used, the SIR model does not convey all real-world spreading scenarios. In particular, in the SIR model, a node can infect its neighborhood several times. In other words, a node has many chances to infect or influence its neighbor(s) before it is removed from the network. Nevertheless, sometimes the diffusion of a disease or a piece of information can be spread by a node only once. That is to say, a node has a single chance to influence its neighbor(s). For instance, consider people meeting in a manifestation. They will meet in this manifestation once, and they may not meet again afterward. The piece of information from one person to another will be transmitted given this one-time chance. Another example is that a person may change his/her opinion towards a cause only if a sufficient number his/her neighbors adopts this opinion. The presence of various conditions that can occur in nodes or edges in the real world necessitates the creation and evaluation of multiple diffusion models.

This allows us to formally pose the main research question of this article: how does the diffusion models’ output depend on the seeds and the network? The seeds are selected based on the community-aware centrality measures. The community-aware centrality measures rely on the network’s structure. The model’s output (i.e., the diffusion spread) depends on the network’s structure and the seed nodes. Thus, we investigate the interplay between the spread of various diffusion models, initiated through the seed nodes selected by the community-aware centrality measures, and the network’s structure. This problem is relevant to many disciplines, from biology and epidemics to sociology and economics. In addition to the diffusion models, there are insufficient studies using a multiple-spreading phenomenon under a spreading scenario rather than in immunization. Another issue is that the community structure changes using one community detection algorithm over the other, which may impact the diffusion dynamics. This also poses a challenge on how the spread will evolve, given that the same seed nodes initiate the diffusion. Finally, we do not have a clear idea of how the community-aware centrality measures compare in controlled synthetic networks and diverse real-world networks. Indeed, previous works mainly focused on a small set of synthetic and real-world networks. This does not enable us to rigorously answer when community-aware centrality measures outperform and what their bottlenecks are.

All of the stated challenges are tackled in this article. To conduct the study, we systematically use eight community-aware centrality measures on a set of four conceptually different diffusion models using a set of synthetic and real-world networks from diverse domains under the multiple-spreader scheme. Therefore, three main parameters are under investigation: the diffusion model and its underlying dynamics, the network and its topological characteristics, and the community-aware centrality measures and their inner workings. The employed diffusion models are the Susceptible-Infected (SI) model, Susceptible-Infected-Recovered (SIR) model, the Linear Threshold (LT) model, and the Independent Cascade (IC) model. Synthetic networks are generated using the LFR algorithm [13] where several parameters can be varied, including the community structure strength, the community size distribution, and the degree distribution. In real-world networks, Infomap and Louvain community detection algorithms are used to uncover the underlying community structure, and their impact is also studied. The community-aware centrality measures and their time complexities as well as the networks are discussed in more detail in S1 Text.

In this article, we contribute to the literature by:

1. Enhancing comprehension of the spread of node influence across diverse diffusion models and network structures.
2. Highlighting how the network structure and budget availability can impact the selection of seed nodes based on community-aware centrality measures.
3. Providing a solid outset for practitioners to select seed nodes that maximize diffusion based on the network structure, budget availability, and the diffusion model that applies in their research case.

## 2 State of the art

Influential nodes are critical in boosting or curbing spreading phenomena in complex networks. A multitude of classical centrality measures has been proposed to quantify node influence. These measures prove their merit in many scenarios, like assessing the infectious capacities of nodes [14] to quantifying financial distress [15] and applying viral marketing [16]. Researchers have shown that classical centrality measures may undermine the influence of nodes in networks with community structure [4–11]. Indeed, many real-world networks are characterized by a community structure that drastically impacts spreading dynamics [1–3]. Thus, in networks with a community structure, nodes that may not be considered influential by a classical centrality measure (i.e., agnostic about the community structure) may be of ultimate influence when one considers the mesoscopic organization of the network.

The exploitation of communities to identify influential nodes using centrality dates back to 2005 when Guimerà and Amaral [4] proposed Participation Coefficient, which uncovered key metabolites across species in metabolic networks. Zhao *et al*. [5] proposed Community-based Centrality, capable of identifying influential nodes in which the classical degree, betweenness, and eigenvector centralities could not identify in the Susceptible-Infected-Recovered (SIR) model with a single-spreader scheme. Unlike Community-based Centrality, Comm centrality proposed by Gupta *et al*. [6] adapts to the network’s strength of community structure, succeeding in identifying hubs and bridges, with the latter being prioritized in an immunization scenario using SIR. Luo *et al*. [7] merged the network’s community structure and hierarchy to develop K-shell with Community, proving its outperformance against classical centrality measures in the SIR model with a single-spreader scheme. Tulu *et al*. [8] showed that using the entropy of a node’s intra-community and inter-community links, nodes disseminating information quickly can be better identified in the SIR model. Ghalmane *et al*. [9] proposed Community Hub-Bridge, which showed its effectiveness in hindering an epidemic by immunizing influential nodes under the SIR dynamics in networks with a strong community structure. Magelinski *et al*. [10] exploited the so-called modularity, a quality measure to assess the community structure of a network, to identify hubs and bridges. The authors showed that their community-aware centrality could dismantle a very large infrastructural network eight times more effectively than other centrality measures by taking a limiting case of the SIR model. Recently, Blöcker *et al*. [11] showed the merit of an information-theoretic community-aware centrality measure based on the map equation in the SIR model using a single-spreader scheme and the Linear Threshold (LT) model using a multiple-spreader scheme.

**Fig 1:**
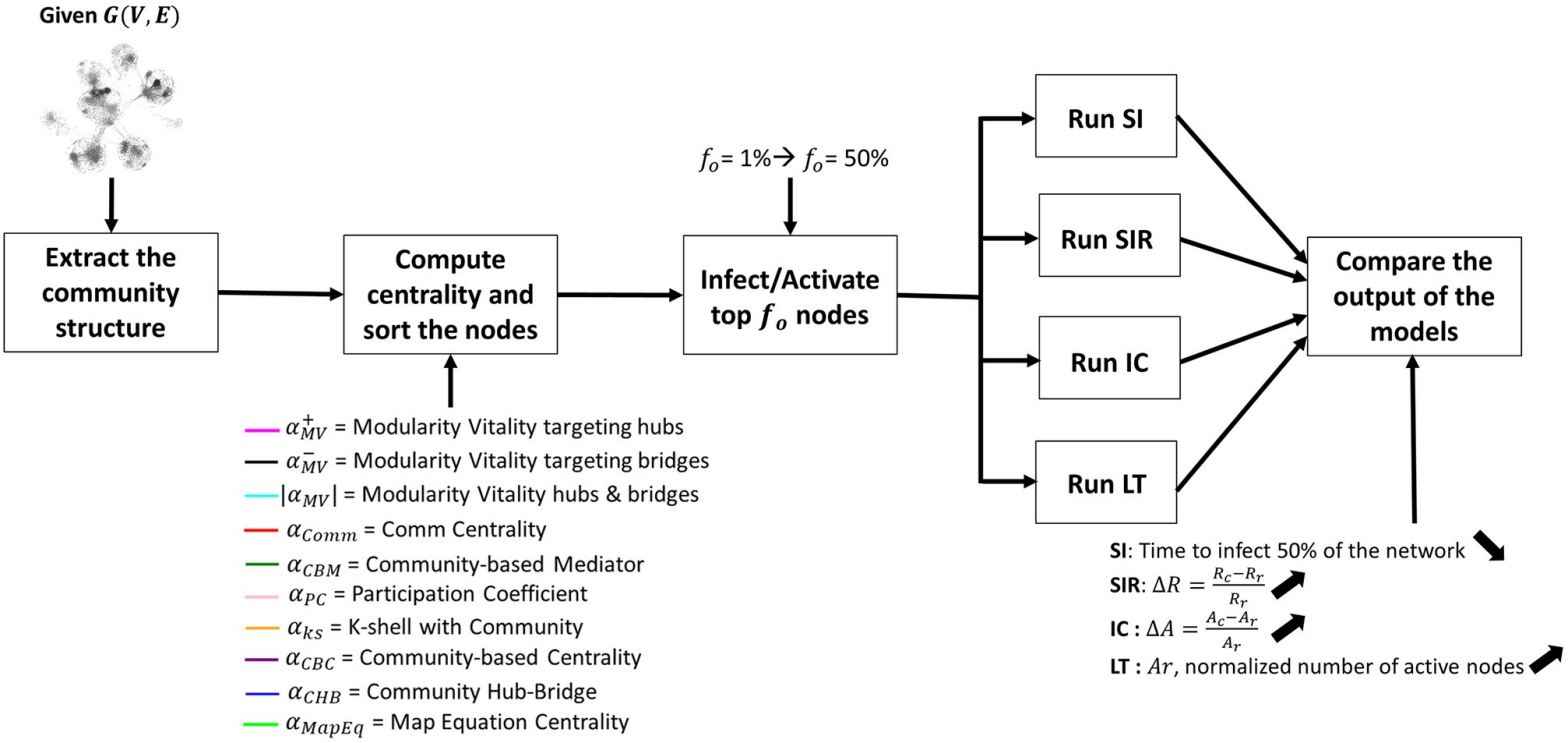
The main steps in our study. First, we extract the community structure if it is not known, then we compute the community-aware centrality measures and sort the nodes from most influential to least. Afterwards, we take a top fraction of nodes denoted as *f*_*o*_ to be infected/activated. The size of *f*_*o*_ varies according to a predefined budget, which we set from 1% of the network’s size to 50% in our study. Then, these selected nodes will initiate the dynamics under the four conceptually different models: the SI, SIR, IC and LT models. When the models reach their stable state, we compare their output. Every model has a different evaluation measure to evaluate the output. In the SI model, the average number of iterations needed to infect 50% of the network is computed: the lower the number of iterations, the more effective the centrality measure. In the SIR and IC models, the relative outbreak and activation size denoted as Δ*R* and Δ*A*, respectively are computed. This value quantifies the difference between the number of nodes recovered or activated based on a given community-aware centrality measure and a reference measure which is the classical degree centrality in our case: the higher Δ*R* and Δ*A* are, the better the performance of the community-aware centrality measure. Finally, in the LT model, the evaluation measure is the total number of activated nodes normalized by the size of the network: the higher *Ar* is, the better.

Despite the outperformance of the community-aware centrality measures compared to classical ones in identifying influential nodes, several limitations need to be addressed. First, most of the community-aware centrality measures are majorly assessed under the SIR dynamics, either to maximize diffusion [5, 7, 8, 11] or minimize it [6, 7, 9, 10] by removing the most central nodes. The latter case is also referred to as “immunization.” Nevertheless, the SIR model does not characterize all situations. Despite researchers’ aim to develop generalized diffusion models, many cases entail adding specific conditions that are not applicable in all real-world situations. Therefore, many diffusive models exist to characterize better cases occurring in the real world. Only one community-aware centrality measure, the Map Equation Centrality [11], is assessed using the SIR and LT dynamics. Second, most of the studies maximizing SIR diffusion use the single-spreader scheme. That being said, Participation Coefficient [4] is the only community-aware centrality measure not assessed with respect to a dynamic model since its original aim was to identify key proteins and construct cartography of metabolic networks rather than analyze the measure’s diffusive power. Third, many studies use a single community detection algorithm if the network’s community structure is unknown. Therefore, it is not well understood how the mesoscopic arrangement of communities affects the dynamics within the same network. Finally, every community-aware centrality measure is assessed on a small sample of real-world and synthetic networks. Table 1 summarizes the works concerning the development and comparison of community-aware centrality measures. The limitations raise several concerns regarding the consistency of the community-aware centrality measures, and this article aims to address these questions. Fig 1 illustrates the methodological process followed in this article. The code and the datasets are accessible via GitHub: https://github.com/StephanyRajeh/DiffusionDynamicsAndCommAwareCentrality.

**Table 1:** A summary of the studies of community-aware centrality measures. SIR means Susceptible-Infected-Recovered model and LT refers to Linear Threshold model. The character ‘-’ refers to “not applicable”. ↘ indicates that the goal is to minimize diffusion and ↗ indicates that the goal is to maximize diffusion.

Community-aware	Diffusion model	Node selection	Real-world	Synthetic	Number of community
centrality measures	& goal	method	networks	networks	detection algorithms
Participation Coefficient [4]	-	-	12	-	1
Community-based Centrality [5]	SIR ↗	Single	6	-	5
Comm Centrality [6]	SIR ↘	Multiple	4	3	1
K-shell with Community [7]	SIR ↗ ↘	Single & Multiple	4	-	1
Community-based Mediator [8]	SIR ↗	Single & Multiple	5	2	1
Community Hub-Bridge [9]	SIR ↘	Multiple	6	5	3
Modularity Vitality [10]	SIR ↘	Multiple	2	3	1
Map Equation Centrality [11]	SIR ↗ & LT ↗	Single & Multiple	12	1	2

## 3 Diffusion models

Diffusion in complex networks is an important interdisciplinary research area representing many real-world situations. Researchers from various domains were attracted to developing models for a more realistic characterization of dynamics on networks. The goal is to describe the current dynamic situation better to apply well-informed decisions and predict future trends. For instance, models were proposed to combat malware spreading across computer networks [17], to optimize online marketing campaigns [18], and to forecast COVID-19 at different territorial levels [19]. Thus, it is clear that one model characterizing all real-world situations is insufficient.

Due to the ubiquity of dynamic interactions across networks in many domains, there is a rich taxonomy for diffusion models. Some researchers refer to them as simple and complex contagions [20–22]. The dynamics of a simple contagion designate that a single contact with an active/infected node is enough for successful transmission. With a complex contagion, a node requires an aggregation of connections with its neighborhood for successful communication to take place. Other researchers divide diffusion models into biological/epidemic models and social/information diffusion models [23–26].

Epidemic models characterize the spread of a virus between individuals, with various parameters in place, such as the infection rate and the recovery rate. In information diffusion, the goal is to simulate the influence of one person over others through passing knowledge, ideas, or opinions toward products or controversial topics. Diffusion models also can be divided into explanatory and predictive models [27–29]. In explanatory models, given an ordered sequence of activated nodes, the goal is to backtrack the evolution of the propagation. In predictive models, the aim is to infer the development of the diffusion process from a set of activated nodes. One can further divide predictive models into graph-based and non-graph-based [27].

Regardless of the taxonomy, popular models mainly differ in four main aspects. The first is the number of states a node can acquire. For instance, in the Susceptible-Infected-Recovered (SIR) model [30], a node can be in one of three states. In contrast, in the Susceptible-Infected (SI) model [30], the node can be either susceptible or infected. The second is the frequency of an activated node capable of influencing other nodes. In the Independent Cascades (IC) model [31], an activated node has a single chance of affecting its neighboring nodes. On the contrary, in the Linear Threshold (LT) model [32], more than one possibility of activation is possible. The third main difference relates to the conditions set on nodes and/or edges. For example, in the SIR model, a constant infection rate is set, while in the IC model, the probability of influencing neighboring nodes can vary. Finally, the fourth main difference is whether the model follows the simple contagion or complex contagion dynamics: the dynamics of a simple contagion indicate that a single contact with an active/infected node is enough for successful transmission. While with a complex contagion, a node requires an aggregation of connections with its neighborhood for successful transmission to take place. Note that one can use the terms active/infected and inactive/susceptible interchangeably.

We are interested in using various models to study the interplay of the diffusion process and the networks given a set of activated nodes selected based on the community-aware centrality measures. In this study, we consider four diffusion models:

- The Susceptible-Infected (SI) model
- The Susceptible-Infected-Recovered (SIR) model
- The Linear Threshold (LT) model
- The Independent Cascade (IC) model

**Fig 2:**
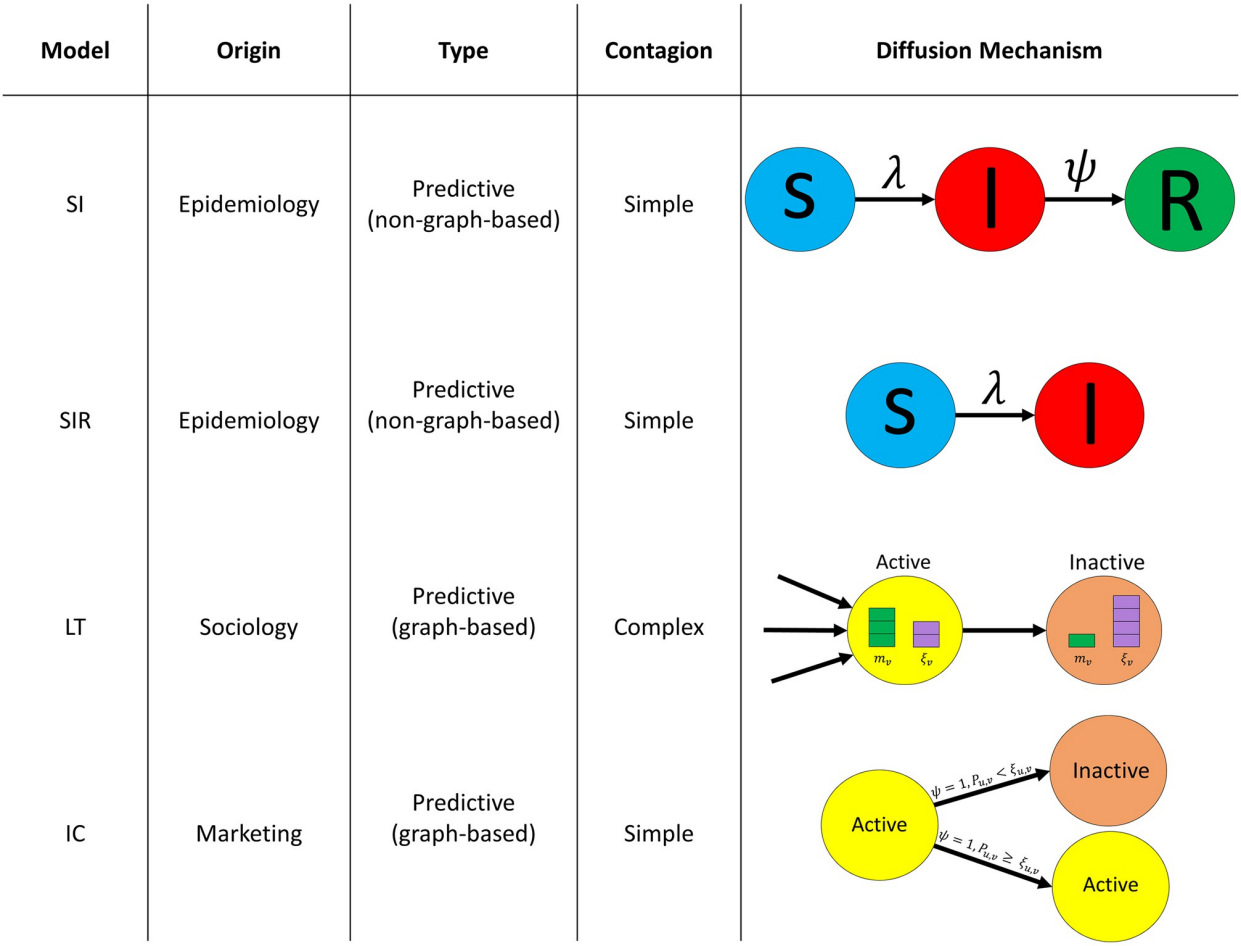
Comparing the diffusion models under study. λ is the infection rate, *ψ* is the recovery rate, *m*_*v*_ is the total number of active neighbors node *v* possesses, *ξ*_*v*_ is node the threshold of node *v*, *P*_*u*,*v*_ is the likelihood of node *u* activating node *v*, and *ξ*_*u*,*v*_ is the threshold of edge (*u*, *v*).

We choose these models for three main reasons: their popularity in the scientific community, their capacity to model realistically diverse diffusion phenomena, and their genericity. The SI and the SIR models originate from epidemiological modeling, while the LT and IC models originate from information diffusion modeling. Additionally, the SI, SIR, and IC models are simple contagion processes where an active node has a single chance of activating another node. In this case, an inactive node does not rely on collective influence to change its state. A single event from an influential activated node is enough for it to become active. In contrast, in the LT model, the success of a transmission depends on the aggregation of the activations of a node’s neighborhood. Finally, all these models are predictive since they all predict the diffusion spread in a network given a set of activated nodes. Fig 2 illustrates the main characteristics of the four models. In the following sections, we discuss each model in more detail.

We note that in this study we are not addressing the problem of influence maximization (i.e., finding the smallest set of nodes that ignites the maximal activation size of nodes). Instead, we are more interested in the interplay between the dynamic models, the network structure, and seed nodes selected using centrality measures with different budget availabilities. The influence maximization problem is NP-hard. Nevertheless, several algorithms have been proposed to approximate this problem [33, 34]. In addition to the research conducted on the differences between simple and complex contagions, network dynamics can also be studied from a resiliency point of view. In that matter, Gao, Barzel, and Barabási [35] explored the dynamics of resilience in complex networks and found a universal resilience pattern across networks, regardless of the network’s structure, that is derived based on the effective state of each node which is in turn affected by the state of its neighbors. Their findings help predict critical points in any network and thus set proactive measures to deter possible perturbations and thus render a resilient system. For more information about influence maximization, one can refer to [36, 37].

## 4 Diffusion dynamics in synthetic networks

This section investigates the interplay between the network’s community structure, the various diffusion mechanisms based on the four models under study (i.e., SI, SIR, IC, and LT), and community-aware centrality measures on a set of synthetic networks generated by the Lancichinetti, Fortunato, and Radicchi (LFR) algorithm [13]. This algorithm allows the tuning of various parameters of the community structure. We investigate the influence of the community structure strength controlled by the mixing parameter (*μ*), the degree distribution power-law exponent (*γ*), and the community size distribution power-law exponent (*θ*). These parameters were chosen based on their significant impact on network structure, the community structure strength *μ*, which has been extensively studied [13, 38, 39]. More details about the synthetic networks and the parameters set to generate them are provided in S1 Text. We note that the networks studied in this article are considered simple, undirected, and unweighted.

### Influence of the community structure strength

**Fig 3:**
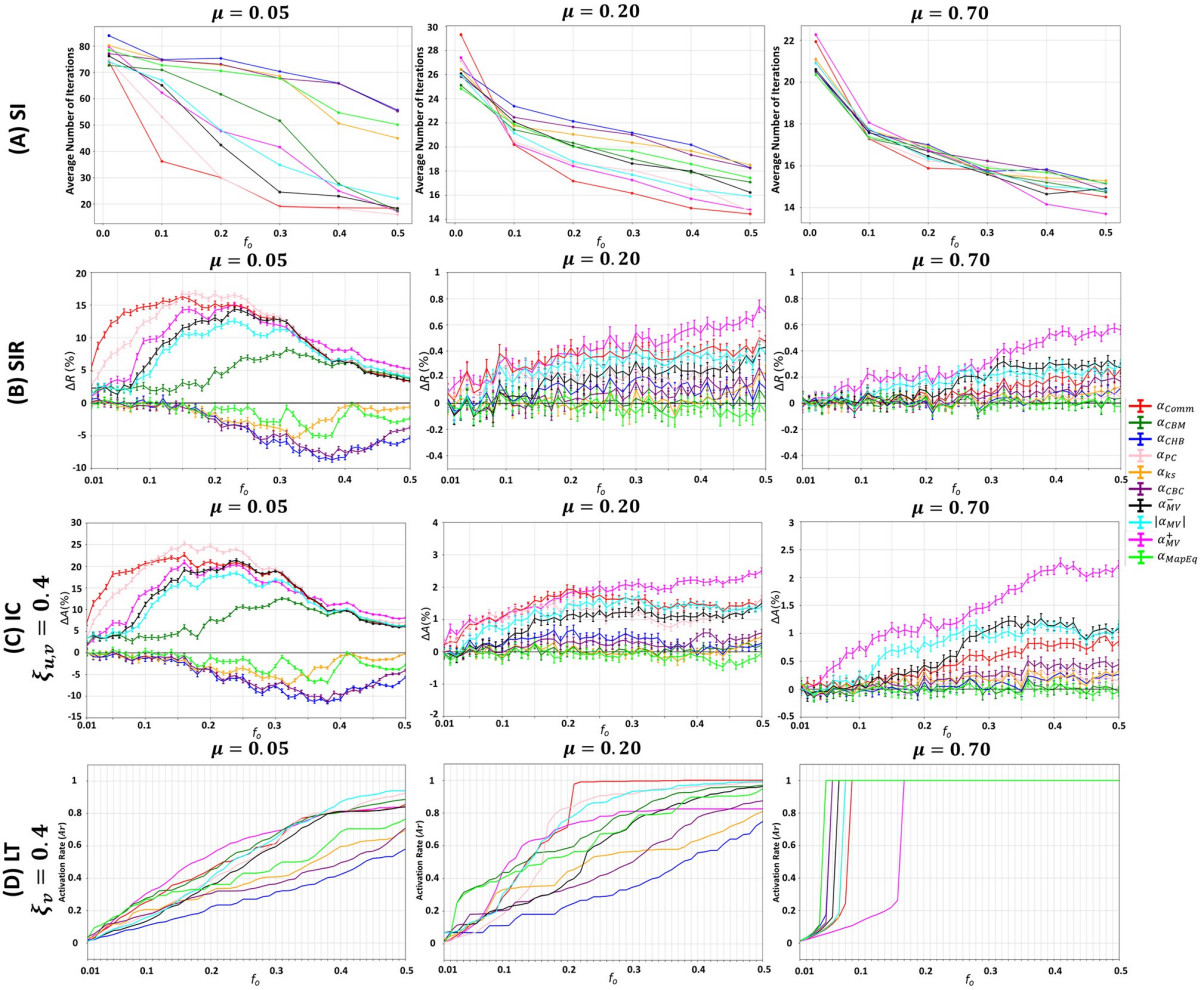
Behavior of the community-aware centrality measures under various dynamic models in synthetic networks while varying the mixing parameter (*μ*). The first, second, third, and fourth rows indicate the results of the (A) SI model, (B) SIR model, (C) IC model, and (D) LT model.

The mixing parameter (*μ*) controls the community structure strength. Low values yield networks with a strong community structure since few inter-community links exist. As *μ* increases, the network’s community structure strength loosens, resulting in the disappearance of dense and well-defined regions. To study the effect of the community structure strength on the various diffusion dynamics, we generate LFR networks with strong (*μ* = 0.05), medium (*μ* = 0.20), and weak (*μ* = 0.70) community structures. Given the ranking of a community-aware centrality measure, a fraction *f*_*o*_ of the top-ranked nodes in the network is initially infected/activated in each of the SI, SIR, IC, and LT models. The results are reported in Fig 3. The evaluation measure in the SI model is the average number of iterations needed for a given *f*_*o*_ to infect 50% of the network. The lower the number of iterations, the more effective the centrality measure. In the SIR and IC models, the relative outbreak/activation size (i.e., Δ*R*/Δ*A*) is computed. This value quantifies the difference between the number of nodes recovered/activated at the end of the dynamical process when *f*_*o*_ is based on a given community-aware centrality measure and a baseline measure which is the degree centrality. Recall that $\Delta R=\frac{R_{c}-R_{r}}{R_{r}}$ (see S1 Text for more details). The higher it is, the better the performance of the community-aware centrality measure. Finally, in the LT model, the evaluation measure is the total number of activated nodes normalized by the size of the network (i.e., the activation rate *Ar*).

Two main phenomena dominate as the community structure strength (*μ*) varies from strong (*μ* = 0.05) to weak (*μ* = 0.70). First, the stronger the community structure, the more pronounced the difference is in the performance of the community-aware centrality measures. As the community structure strength decreases (i.e., weakens), the performance of the community-aware centrality measures becomes more comparable, and differences are less visible. Community-aware centrality measures are well-adapted to networks with a well-defined community structure. With this structure, each measure can exploit various community information to identify influential hubs and bridges that contribute to the network’s community structure. If the community structure is loosely defined, it becomes more difficult for the community-aware centrality measures to pinpoint these influential nodes. Indeed, in a weak community structure, hubs and bridges become less prominent, and the average degree of the nodes becomes more analogous.

The second phenomenon is related to the divergence in the scales (i.e., the magnitude of the evaluation measures) while the dynamical processes take place. In the SI model (Fig 3A), with a strong community structure, the epidemic diffusion needs more iterations to reach 50% of the network. As communities share a few inter-community links in a network with a strong community structure, the infection tends to stay more localized in the communities. With a decrease in the community structure strength, the proportion of inter-community links increases. Therefore, the infection can spread more quickly to the remaining communities. Thus, fewer iterations are needed to infect half of the network. In the SIR and IC models (Fig 3B and 3C), it is clear that when the network has a strong community structure, a set of community-aware centrality measures outperform degree centrality by a large difference. However, as the community structure strength weakens, the community-aware centrality measures become more comparable to the performance of the degree centrality.

Inspecting the community-aware centrality measures in more detail, most of these measures are a variant of degree centrality exploiting the inter-community and intra-community links in various ways. The smaller the difference between these two types of links—usually prevailing in a network with a loose community structure—the higher the resemblance of the community-aware centrality measures to degree centrality. Therefore, in a network with a weak community structure, the outperformance of the community-aware centrality measures is insignificant compared to degree centrality. The latter is advised for usage as it does not need community-level information. Nevertheless, with a strong community structure strength, community-aware centrality measures can extract information that the community-agnostic degree centrality cannot.

In LT model (Fig 3D), when the network has a strong community structure strength, achieving full activation of nodes is unattainable, even with an initial activation fraction (*f*_*o*_) set at 50%. Nevertheless, when the community structure weakens, lower values of *f*_*o*_ can result in complete activation, particularly when *μ* is set at 0.7. This suggests that in networks with reduced community cohesion, the diffusion of activations becomes more and easily achievable.

An important finding can be extracted independently of the community structure strength (*μ*). It concerns the similarities of the various dynamic models. By visually inspecting Fig 3, one can note that generally, at any given *f*_*o*_, the top 2 outperforming centrality measures are comparable across the SI, SIR, and IC models, excluding the LT model. For instance, at *f*_*o*_ = 0.05 and *μ* = 0.05, the top 2 outperforming centrality measures are Comm Centrality (*α*_*Comm*_) and Participation Coefficient (*α*_*PC*_) in the SI, SIR, and IC models. In contrast, Map Equation Centrality (*α*_*MapEq*_) and Community-based Mediator (*α*_*CBM*_) are the top 2 most performing in the LT model at *f*_*o*_ = 0.05 and *μ* = 0.05. This behavior is logical as the SI, SIR, and IC are a variant of one another. The SIR is the SI with an additional “recovered” state. The IC sets thresholds on edges, and nodes have one chance to infect/activate their neighbors, while in the SIR model, a node has more than one chance. Even though differences exist, they are nominal. Indeed, their dynamics follow the simple contagion dynamics where nodes getting activated/infected are independent of their surroundings. This is not true for the LT model, where a node’s activation depends on its neighborhood’s aggregate activations. Subsequently, activations are harder to diffuse across the network, especially if the network has a strong community structure strength [40, 41].

These results suggest that the community-aware centrality measures are more profitable in networks with a strong community structure strength. They also suggest that one should be prudent in using the measures even with a strong community structure strength, as the outperformance depends on the model. Some measures are well-suited to the SI, SIR, and IC models, while others are more suited to the LT dynamics.

### Influence of the community size distribution exponent

**Fig 4:**
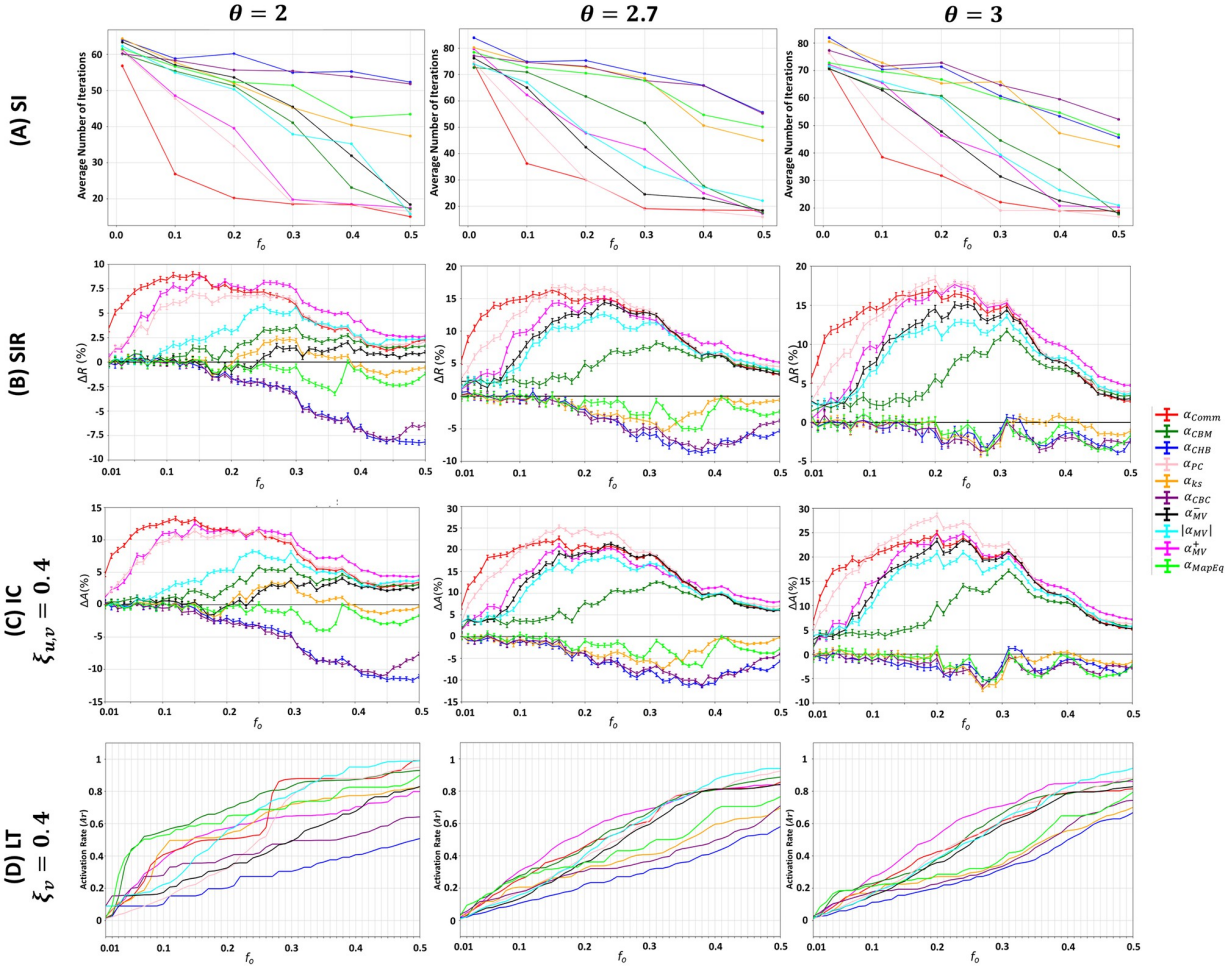
Behavior of the community-aware centrality measures under various dynamic models in synthetic networks while varying the community size distribution exponent (*θ*). The first, second, third, and fourth rows indicate the results of the (A) SI model, (B) SIR model, (C) IC model, and (D) LT model.

The community size distribution exponent (*θ*) is responsible for the frequency and the size of the generated communities. We fix the community structure strength at *μ* = 0.05 and generate three networks with three different community size distribution exponents. The first, having *θ* = 2, indicates that large communities make up most of the network, with the existence of few small communities, resulting in a large variance in the community sizes. The second, having *θ* = 2.7, yields less variance in the community sizes with a larger number of communities. Finally, the third, having *θ* = 3, a high number of communities exist with equivalent sizes. Given the ranking of a community-aware centrality measure, a fraction *f*_*o*_ of the top-ranked nodes in the network is initially infected/activated in each of the SI, SIR, IC, and LT models. The results are reported in Fig 4.

In case the dynamics follow the SI, SIR, or IC, illustrated in the first three rows of Fig 4, it can be noticed that the general trends of the community-aware centrality measures persist whether the network is generated with *θ* = 2, *θ* = 2.7, and *θ* = 3. The main difference is in the magnitude of the final output of each of the models. However, with the LT model, illustrated in the last row of Fig 4, the behavior of the community-aware centrality measures changes with every *θ* under investigation.

Particularly, in the SI model, in the network with a larger variance in the community size distribution (i.e., at *θ* = 2), it takes less time to infect 50% of the network compared to networks with fewer communities of equivalent sizes. For instance, when *θ* = 2, at *f*_*o*_ = 0.2, the average number of iterations for Comm Centrality (*α*_*Comm*_), the best performing centrality, takes 20 iterations while with *θ* = 2.7, it takes 30 iterations and with *θ* = 3 it takes 32 iterations. The magnitude of the relative outbreak size (Δ*R*) in the SIR model shows that the outperformance of Comm Centrality (*α*_*Comm*_), Modularity Vitality targeting hubs ($\alpha_{MV}^{+}$), and Participation Coefficient (*α*_*PC*_) is more pronounced in the networks with *θ* = 2.7 and *θ* = 3. For instance, let’s take *f*_*o*_ = 0.10, Δ*R* of Comm Centrality amounts to 8.5% in the network with *θ* = 2, while in the networks with *θ* = 2.7 and *θ* = 3, Δ*R* amounts to 15%. Under the IC dynamics, the highest magnitude of the relative activation size (Δ*A*) is reached when *θ* = 2 (Δ*A* = 13%). In contrast, when *θ* = 2.7 and *θ* = 3, the maximum Δ*A* reached amounts to 25% and 28.5%, respectively.

Despite the similarities in the general trends of the community-aware centrality measures, one subtle difference needs to be noted concerning Participation Coefficient. In the three models, Participation Coefficient performs less when *θ* = 2. As stated earlier, *θ* = 2 indicates that the network is characterized by a few small communities and many large communities, which comprise most of the network. Participation Coefficient’s effectiveness depends on the number of communities within the network. If there are many communities, it has more room to distinguish the difference in influence between nodes. On the other hand, having fewer communities makes it less effective as many nodes will have similar centrality, making it difficult to distinguish their influence characteristics.

In the LT model, the impact of adjusting *θ* is more pronounced on the performance of the top-performing community-aware centrality measures in terms of behavior rather than magnitude. The contrast becomes more noticeable when the budget range goes from low to medium. When the budget is high, in all of the studied *θ*, the strategy is to target hubs and bridges together using Modularity Vitality targeting hubs and bridges (|*α*_*MV*_|). We now discuss when the budget spans from low to medium. When the network has a significant variance in the community size distribution (*θ* = 2), hub-like nodes are preferred up to a small value for the budget availability, then bridge-like nodes are preferred. In contrast, with a smaller variance in the community size distribution (*θ* = 2.7 and *θ* = 3), hub-like nodes are always preferred. More specifically, when the value of *θ* is equal to 2, the hub-like nodes that produce the highest outbreak until *f*_*o*_ reaches 0.05 are selected by Map Equation Centrality (*α*_*MapEq*_) and Community-based Centrality (*α*_*CBC*_). From *f*_*o*_ = 0.06 to *f*_*o*_ = 0.34, bridge-like nodes selected by Community-based Mediator (*α*_*CBM*_) and then by Comm Centrality (*α*_*Comm*_) are the nodes that generate the highest activation rate. If *θ* is equal to 2 or 2.7, the hub-like nodes preferred at the small budget are chosen by Map Equation Centrality (*α*_*MapEq*_), and then Modularity Vitality targeting hubs ($\alpha_{MV}^{+}$) takes over for a broader range of budget availabilities.

To sum up, the results of the SI, SIR, and IC models suggest that changing the community size distribution exponent has a greater community-aware centrality measures’ magnitude in the model’s output rather than their behavior. In networks with a large variance in community sizes, the outbreak size in the SIR model and the activation size in the IC model are not as pronounced as in networks with a smaller variance, implying that the outbreak can more easily spread to many communities with equivalent sizes. However, many communities may remain unaffected if the infection starts in big communities and remains within them. In the SI model, when there is a large variance in community sizes, it takes less time to infect 50% of the network since it consists of only a few big communities. Suppose many nodes are in the same community, making up almost 50% of the network. In that case, it is easy to infect/activate that community because the nodes in the community are more likely to be directly or indirectly connected. In the LT model, the community size distribution has a greater effect on the behavior of community-aware centrality measures than their magnitude. When there is a large variance in community size distribution, bridge-like nodes play a crucial role in the medium budget range, as the selected bridges are likely to be located in large communities, resulting in a higher outbreak. Conversely, when there are many communities of similar sizes, it is more beneficial to target hub-like nodes since the influence of bridge nodes may stop at the border of a community with high density. These findings are supported by studies on contagions and networks [40, 41].

### Influence of the degree distribution exponent

**Fig 5:**
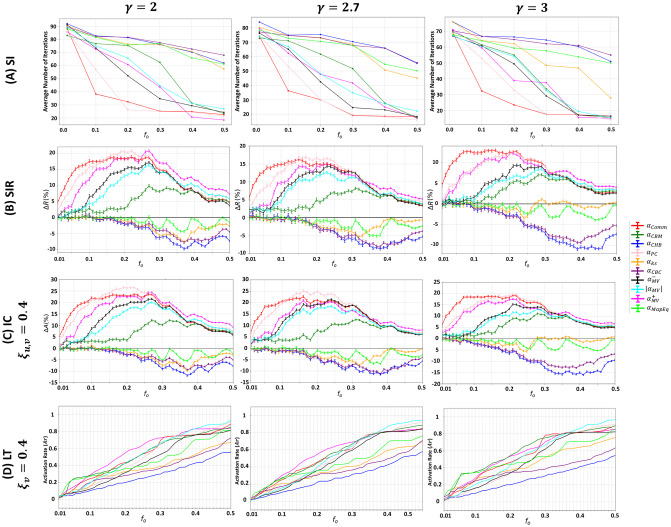
Behavior of the community-aware centrality measures under various dynamic models in synthetic networks while varying the degree distribution exponent (*γ*). The first, second, third, and fourth rows indicate the results of the (A) SI model, (B) SIR model, (C) IC model, and (D) LT model.

The degree distribution exponent (*γ*) characterizes the number of links nodes have in a network. The LFR algorithm generates networks with a power-law degree distribution fitting the degree distribution of many real-world networks [42, 43]. Many real-world networks are distinguished by *γ* falling between 2 and 3 [44, 45]. To investigate three representative cases, we fix the community structure strength at *μ* = 0.05 and generate three networks with *γ* = [2, 2.7, 3]. While preserving the community structure, the network portrays a hub-and-spoke structure when *γ* = 2 [46]. On the other extreme, the nodes inside the communities have more comparable degrees, resembling a random-like network when *γ* = 3. At *γ* = 2.7, the network resembles a typical scale-free network. Given the ranking of a community-aware centrality measure, a fraction *f*_*o*_ of the top-ranked nodes in the network is initially infected/activated in each of the SI, SIR, IC, and LT models. The results are reported in Fig 5.

Similar to the variation of the community size distribution exponent (*θ*), the general trend persists when varying the degree distribution exponent (*γ*) in the SI, SIR, and IC models. Indeed, the difference is attributed to the magnitude of the SI, SIR, and IC models’ output, illustrated in the first three rows of Fig 5. While with the LT model, illustrated in the last row of Fig 5, the behavior of the community-aware centrality measures is what changes rather than the magnitude.

To begin with the SI model, the time it takes to infect 50% of the network decreases as the initial fraction of infected nodes (*f*_*o*_) increases, as *γ* increases. Indeed, the network structure impacts the number of iterations it takes to infect 50% of the network. The random-like structure inside the communities, as found in *γ* = 3 networks, results in a faster spread compared to the hub-and-spoke-like structure, as found in *γ* = 2 networks and to a lesser extent when *γ* = 2.7. For example, when *f*_*o*_ = 0.1, the best performing centrality in the SI model, Comm Centrality (*α*_*Comm*_), takes an average of 39 iterations to infect 50% of the network in a *γ* = 2 network, 36 iterations in a *γ* = 2.7 network, and 31.5 iterations in a *γ* = 3 network. In the SIR and IC models, the output of all the measures performs more in the networks with *θ* = 2 and *θ* = 2.7 compared to *γ* = 3. Let’s take *f*_*o*_ = 0.10 following the SIR model, Δ*R* of Comm Centrality amounts to 17% in the network with *γ* = 2, to 16% in the network with *γ* = 2.7, and to 13% in the network with *γ* = 3. Under the IC dynamics, the maximum relative activation size (Δ*A*) when it equals 2 is 26.5%, while at *γ* = 2.7 and *γ* = 3, the Δ*A* reached is 25% and 19.5%, respectively.

Although there are similarities in the overall patterns of the community-aware centrality measures in the SI, SIR, and LT models, the Participation Coefficient is influenced by changes in the degree distribution exponent, similar to the impact of changes in the community size distribution exponent. Specifically, the Participation Coefficient performs better when the network is created with *γ* = 2 and *γ* = 2.7. This suggests that the Participation Coefficient benefits from having differences in node degrees, which allows it to distinguish between nodes and identify the most influential ones.

The results of the LT model show that the main difference between different values of *γ* is observed when the budget availability is medium. When the budget is high (i.e., *f*_*o*_ ≥ 0.40), targeting hub-like and bridge-like nodes using Modularity Vitality targeting hubs and bridges (|*α*_*MV*_|) is always the most effective strategy, regardless of *γ*. Similarly, when the budget is low (i.e., *f*_*o*_ ≤ 0.05), it is always better to target hub-like nodes selected by Map Equation Centrality (*α*_*MapEq*_). However, when the budget is medium, networks with *γ* = 2 and *γ* = 2.7 tend to benefit more from targeting hub-like nodes using Modularity Vitality targeting hubs ($\alpha_{MV}^{+}$). On the other hand, in networks with *γ* = 3, where the communities are more random, bridge-like nodes become more influential. Community-based Mediator (*α*_*CBM*_) selects nodes that are well-connected between different communities in the network for a higher activation rate in this case.

In brief, results show that community-aware centrality measures exhibit consistent behavior across the SI, SIR, and IC models as the degree distribution exponent changes. However, the models’ output based on these measures varies in magnitude. When the degrees of nodes in communities are similar, the SI model takes less time to infect more of the network. But in the SIR and IC models, community-aware centrality measures are comparable to degree centrality as they are evaluated using the relative outbreak and activation sizes. This is because the measures have less power to differentiate between hub-like and bridge-like nodes when node degrees are similar. In contrast, the LT model’s community-aware centrality measures exhibited differences in behavior rather than magnitude, particularly at medium budget availabilities. Results indicate that targeting bridge-like nodes is better when node degrees are comparable in their communities. This is because similar degrees may imply similar influence, making differentiation difficult. Therefore, selecting bridge-like nodes has a better chance of igniting a more significant impact in the network.

## 5 Diffusion dynamics in real-world networks

In this section, we investigate the interplay between the diffusion dynamics of the SI, SIR, IC, and LT models and the community-aware centrality measures on forty real-world networks. Unlike synthetic networks, the topological characteristics of real-world networks cannot be controlled. Indeed, real-world networks are characterized by diverse structures that affect the diffusion dynamics differently. Moreover, these networks pertain to various domains (i.e., infrastructural, social, acting, biological, and collaborative). Thus, nodes and edges have specific roles in maintaining the normal functioning of the network. Since the community structure of real-world networks is unknown a priori, we uncover their communities using the Infomap [47] community detection algorithm. At a later stage, we investigate the consistency of the results using the Louvain [48] community detection algorithm.

**Fig 6:**
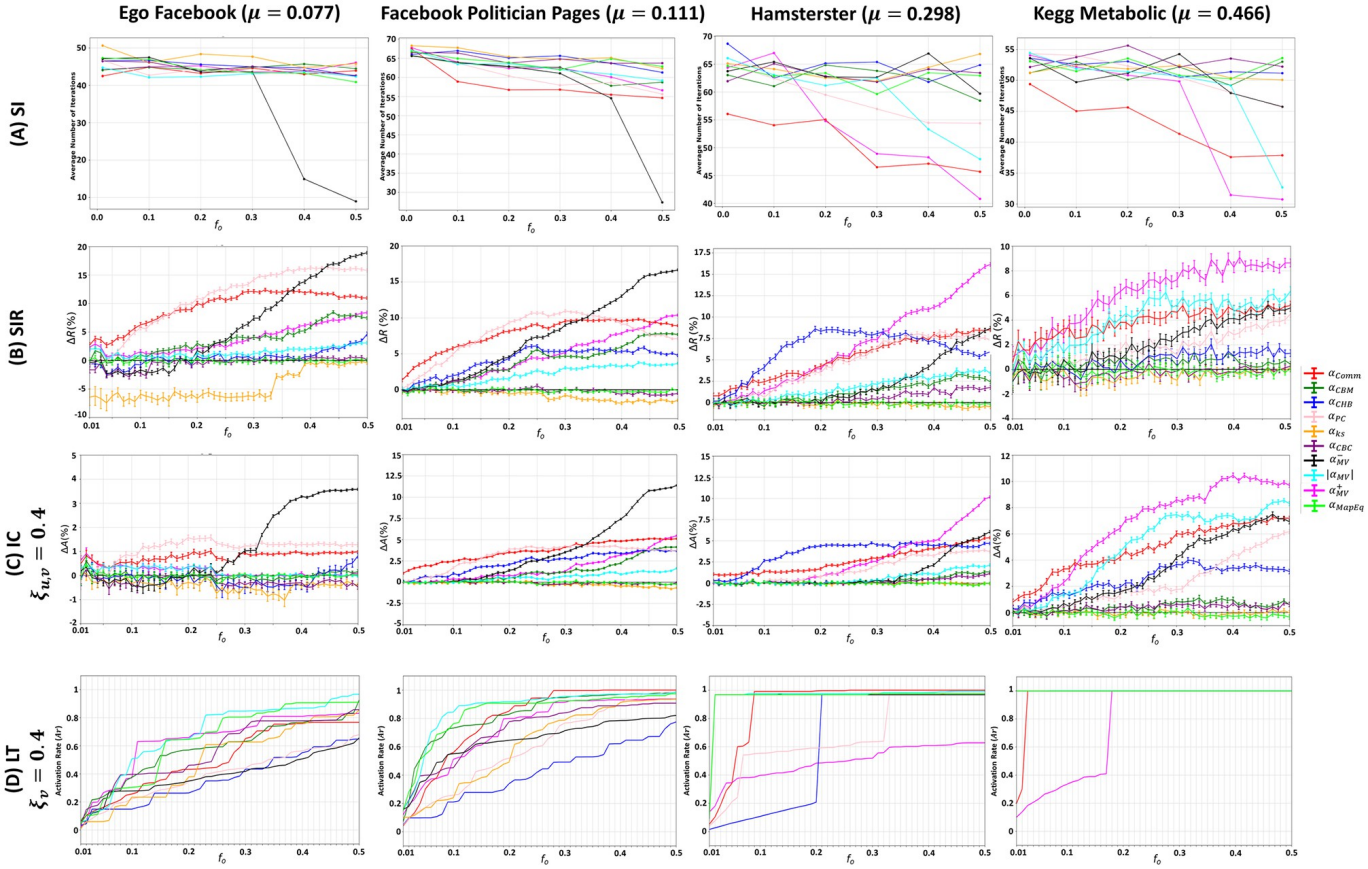
Behavior of the community-aware centrality measures under various dynamic models in real-world networks with varying community structure strengths. The first, second, third, and fourth rows indicate the results of the (A) SI model, (B) SIR model, (C) IC model, and (D) LT model.

Similar to the methodology adopted with synthetic networks, a fraction *f*_*o*_ of the top-ranked nodes in each network is initially infected/activated, given the ranking of a community-aware centrality measure in each of the SI, SIR, IC, and LT models. For brevity, the results of four real-world networks are reported in Fig 6. These networks are representative cases of the dynamics seen across the networks under study. Indeed, the extensive analysis across the models shows two network categories in every diffusion model investigated. These two categories, illustrating a different behavior in terms of the spreading dynamics, can be divided based on the network’s community structure strength. The remaining networks are provided in S1 Text.

The first category comprises networks with medium to weak community structure strengths, such as the networks Hamsterster (*μ* = 0.298) and Kegg Metabolic (*μ* = 0.466). In this category, a shared trend illustrates that up to a certain fraction of initially infected/activated nodes (*f*_*o*_), bridge-like nodes using Comm Centrality (*α*_*Comm*_) outperform the remaining measures. After passing *f*_*o*_, which is network-dependent, hub-like nodes using Modularity Vitality targeting hubs ($\alpha_{MV}^{+}$) outperform other measures in terms of spreading capability in each of the SI, SIR, and IC models. In between, Community Hub-Bridge (*α*_*CHB*_) provides good results in a medium range of *f*_*o*_ only in the SIR and IC models. Results in the LT model diverge from the remaining models. With the LT dynamics, hub-like nodes using Map Equation Centrality (*α*_*MapEq*_) outperform the remaining measures up to a certain *f*_*o*_. Then, it either persists in its outperformance with other measures or Comm Centrality (*α*_*Comm*_), which has a preference for bridge-like nodes, outperforms the remaining measures (as seen with Hamsterster).

The second category comprises networks with a strong community structure strength, such as Ego Facebook (*μ* = 0.077) and Facebook Politician Pages (*μ* = 0.111). Within this category, under the SI, SIR, and IC dynamics, bridge-like nodes always yield the highest performance. The distinction lies in which community-aware centrality measure yields such performance. Generally, Comm Centrality (*α*_*Comm*_) has the highest performance up to a certain *f*_*o*_. Then, Participation Coefficient (*α*_*PC*_) overcomes Comm Centrality (*α*_*Comm*_) only in the SI and SIR dynamics. Afterwards, in the SI, SIR, and IC dynamics, Modularity Vitality targeting bridges ($\alpha_{MV}^{-}$) outperforms all remaining measures. The LT dynamics pose different outcomes. At first, hub-like nodes using Community-based Centrality (*α*_*CBC*_) or Map Equation Centrality (*α*_*MapEq*_) outperform the remaining measures. After exceeding a certain *f*_*o*_, several measures may show high performance, namely Community-based Mediator (*α*_*CBM*_), Modularity Vitality targeting hubs ($\alpha_{MV}^{+}$), Modularity Vitality targeting hubs and bridges (|*α*_*MV*_|), Map Equation Centrality (*α*_*MapEq*_), and/or Comm Centrality (*α*_*Comm*_).

We note that a divergence in scale in the output of the diffusion models occurs among the real-world networks. For instance, the performance of the community-aware centrality measures in networks with a weak community structure is more comparable with degree centrality than in networks with a strong community structure. However, the divergence in scale is less significant than in synthetic networks. Moreover, unlike synthetic networks, differences in the performance of the community-aware centrality measures exist in real-world networks under the SI, SIR, and IC dynamics. A high variance between the curves is seen regardless of whether the network has a strong or weak community structure strength.

The study’s results show that the SI, SIR, and IC models behave similarly to synthetic networks, but the dynamics differ in real-world networks based on the strength of their community structure. In networks with a weak community structure, bridge-like nodes lead to higher outbreaks/activations until hub-like nodes perform better. However, bridge-like nodes always perform better in networks with a strong community structure. Regarding the LT dynamics, hub-like nodes outperform other measures up to a certain *f*_*o*_, and after that, other measures with preferences for hub-like, bridge-like, or both types of nodes show better performance. Additionally, real-world networks have a more pronounced variance between the curves regardless of their community structure strength, showcasing their unique characteristics that diversely affect the network’s dynamics. This contrasts with synthetic networks, where all parameters are controlled.

## 6 Discussion

In this section, we address questions related to why the results of the dynamic models seen with the real-world networks are obtained. We refer to the fraction of initially infected/activated nodes (*f*_*o*_) as “budget availability” thenceforth.

**Fig 7:**
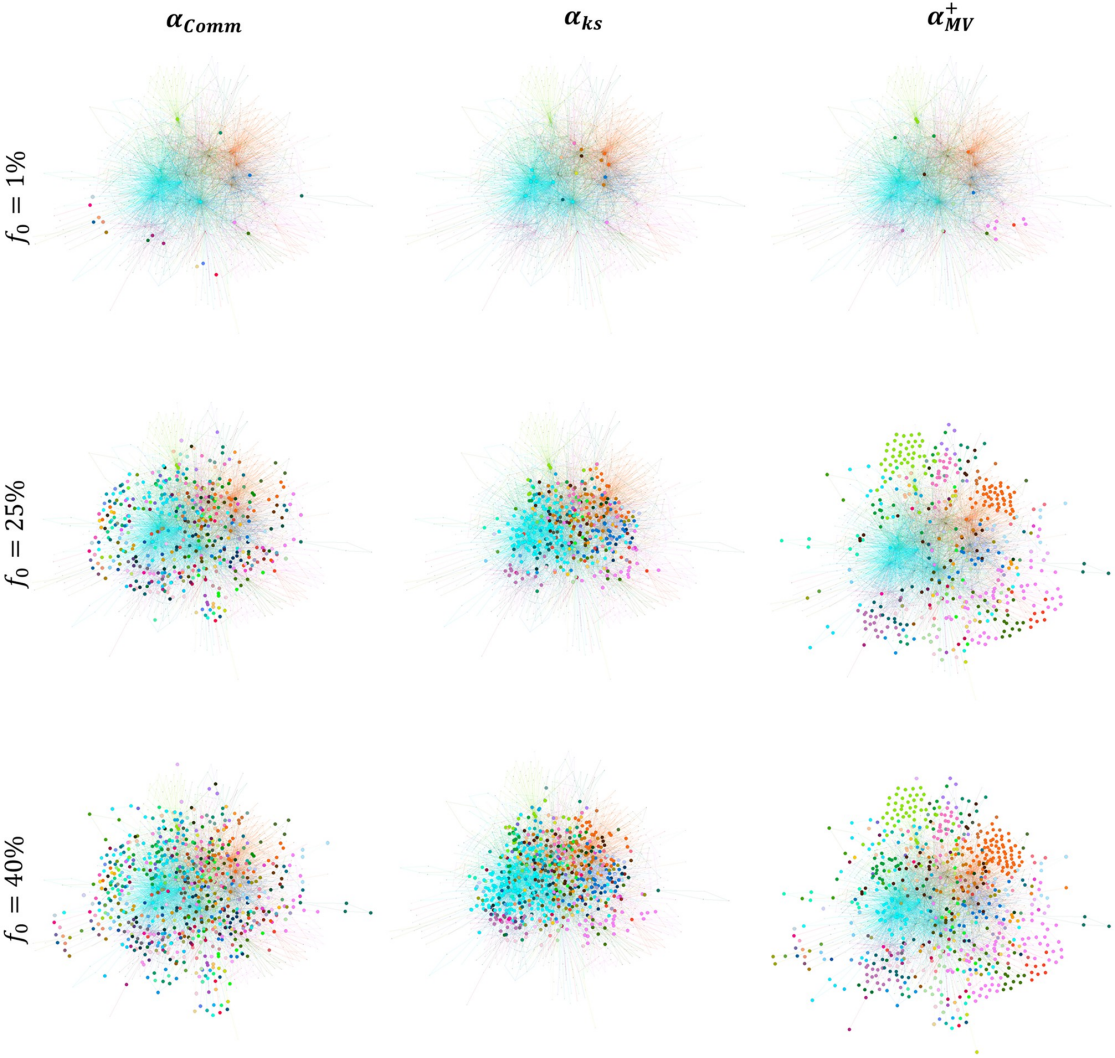
Comparing the position of the top nodes in the Kegg Metabolic network (*μ* = 0.466). The top nodes are chosen at a low budget availability (*f*_*o*_ = 1%), medium budget availability (*f*_*o*_ = 25%), and high budget availability (*f*_*o*_ = 40%). The bigger nodes in the left, middle, and right figures are the top nodes ranked by Comm Centrality (*α*_*Comm*_), K-shell with Community (*α*_*ks*_), and Modularity Vitality targeting hubs ($\alpha_{MV}^{+}$), respectively.

**(1) Why is it more beneficial to target bridge-like nodes at low budget availability and hub-like nodes at high budget availability in the SI, SIR, and IC diffusion models when the network has a medium to weak community structure strength?** As it was previously seen, in networks with a medium to weak community structure strength, Comm Centrality (*α*_*Comm*_) generally results in the highest outbreak when the budget is limited. To investigate why we take the Kegg Metabolic network, which has a weak community structure strength (*μ* = 0.466). Using this network, we compare in Fig 7 the position of the top nodes chosen based on low (i.e., *f*_*o*_ = 1%), medium (i.e., *f*_*o*_ = 25%), and high (i.e., *f*_*o*_ = 40%) budget availabilities. For comparison purposes, we take the various budget availabilities according to the rankings based on Comm Centrality (*α*_*Comm*_), K-shell with Community (*α*_*ks*_), and Modularity Vitality targeting hubs ($\alpha_{MV}^{+}$). As we can see in Fig 7, Comm Centrality (*α*_*Comm*_) targets nodes distributed across the network when the budget is either low (i.e., *f*_*o*_ = 1%) or medium (i.e., *f*_*o*_ = 25%). These nodes yield a higher spreading capability in the SI, SIR, and IC models, as Fig 6 shows.

In contrast, with K-shell with Community (*α*_*ks*_), a measure that generally underperforms in these models, the nodes chosen are close to each other and embedded in the core of the network. Thus, the spreading virus or piece of information will die out before it reaches the peripherical areas in the network. Now, why do hub-like nodes targeted using Modularity Vitality targeting hubs ($\alpha_{MV}^{+}$) at high budget availability yield the highest outbreak in the SI, SIR, and IC models? Referring back to Fig 7, when *f*_*o*_ = 40%, as we can see, the nodes are distributed even more than Comm Centrality (*α*_*Comm*_) across all the regions in the network. Thus, it is normal to have a higher outbreak, as the virus/information would reach all the peripherical areas of the network and its core.

**Fig 8:**
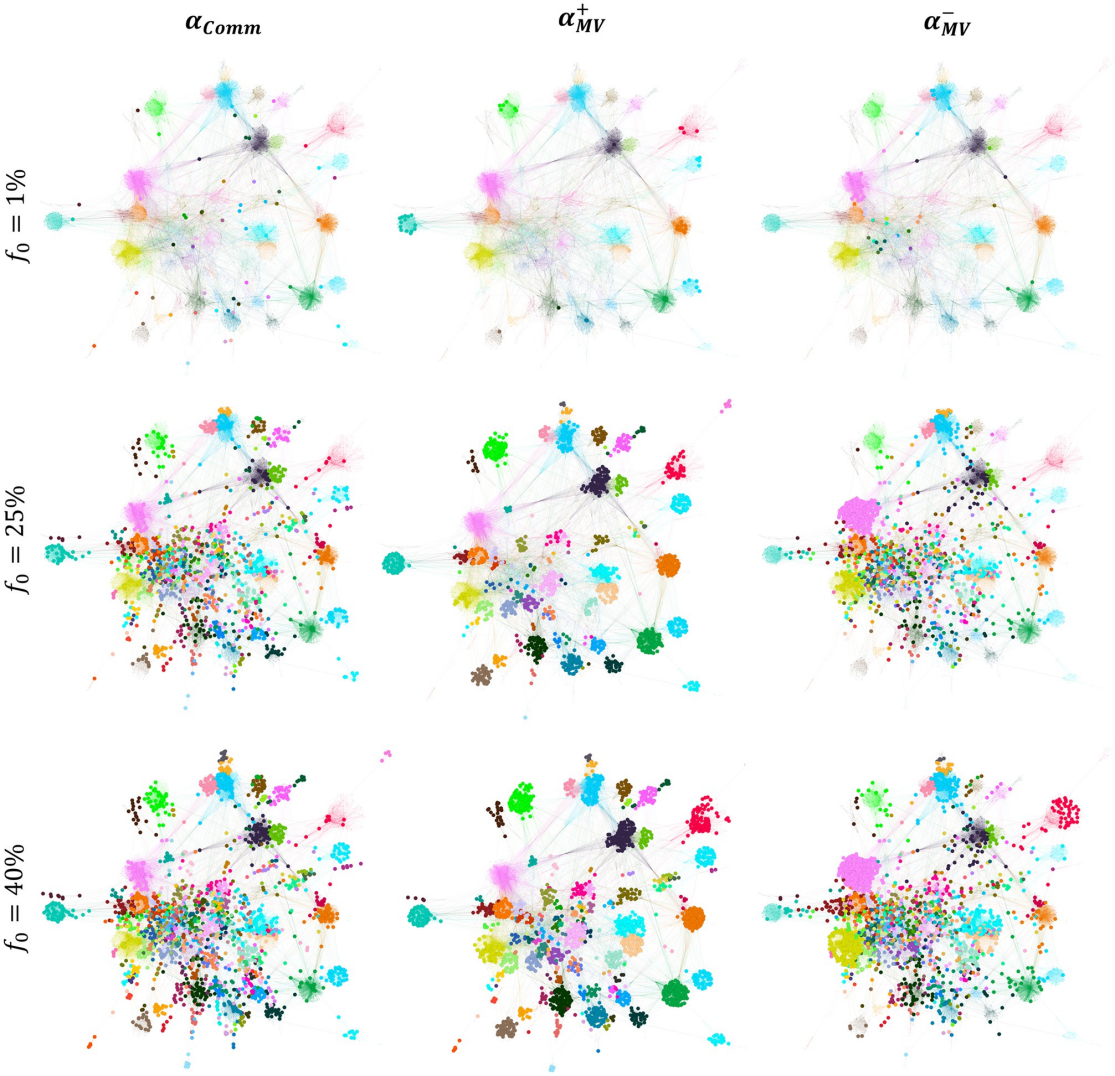
Comparing the position of the top nodes in the Facebook Politician Pages network (*μ* = 0.111). The top nodes are chosen at a low budget availability (*f*_*o*_ = 1%), medium budget availability (*f*_*o*_ = 25%), and high budget availability (*f*_*o*_ = 40%). The bigger nodes in the left, middle, and right figures are the top nodes ranked by Comm Centrality (*α*_*Comm*_), Modularity Vitality targeting hubs ($\alpha_{MV}^{+}$), and Modularity Vitality targeting bridges ($\alpha_{MV}^{-}$), respectively.

**(2) Why is it more beneficial to target bridge-like nodes, regardless of the budget availability, in the SI, SIR, and IC diffusion models when the network has a strong community structure strength?** We have seen that bridge-like nodes always perform well when the network has a strong community structure strength. The distinction lies in which community-aware centrality measure with a preference to bridge-like nodes yields the highest outbreak. The results show that Comm Centrality (*α*_*Comm*_) generally performs best when the budget is limited. Conversely, when the budget availability is high, Modularity Vitality targeting bridges ($\alpha_{MV}^{-}$) overcomes all the measures (see networks Ego Facebook and Facebook Politician Pages in Fig 6). To investigate these results, we visualize in Fig 8 the Facebook Politician Pages network, which has a strong community structure strength (*μ* = 0.111).

For comparison purposes, the top nodes visualized are based on Comm Centrality (*α*_*Comm*_), Modularity Vitality targeting hubs ($\alpha_{MV}^{+}$), and Modularity Vitality targeting bridges ($\alpha_{MV}^{-}$). As we can see, in this network with a strong community structure, when the budget is low (i.e., *f*_*o*_ = 1%) and medium (i.e., *f*_*o*_ = 25%), the top nodes ranked by Comm Centrality (*α*_*Comm*_) are widespread between and across many communities. This spread indicates that the virus/information has many venues to further expand into, permitting a higher outbreak. As the budget increases to 40%, Modularity Vitality targeting bridges ($\alpha_{MV}^{-}$) takes over. The nodes selected by it also spread across many regions in the networks, however, not to the extent of Comm Centrality (*α*_*Comm*_), which reaches even the peripherical communities in the network. Indeed, Modularity Vitality targeting bridges ($\alpha_{MV}^{-}$) focuses on bridges between communities and has a preference to target more nodes inside big communities rather than the peripherical areas. Thus, targeting bridges connecting communities and simultaneously focusing on big communities for higher outbreaks is more effective since small and peripherical communities cannot be leveraged as much as big communities if the budget is high and the network has a strong community structure. Thus, under the SI, SIR, and IC dynamics, choosing nodes inside and between the big communities diffuses the information more widely internally and externally. In contrast, small peripherical communities are isolated and do not have many pathways for the virus/information to spread.

We also shed light on how Modularity Vitality targeting hubs ($\alpha_{MV}^{+}$) behaves. At *f*_*o*_ = 0.40, Modularity Vitality targeting hubs ($\alpha_{MV}^{+}$) does not target the community colored in fuchsia, the biggest community in the network. It does not since a node removed from a big and well-connected community will not change the network’s modularity significantly. In contrast, a hub removed from a smaller community may shatter that community. Consequently, when ranked according to Modularity Vitality targeting hubs, these nodes would receive a higher score ($\alpha_{MV}^{+}$). Thus, having big communities not targeted in a network with a strong community structure yields lower reachability of the virus/information. The behavior is the opposite in a network with a weak community structure. Since all the small communities surrounding a big community will be activated/infected, the infection/information has a higher probability of entering the big community as there are many pathways to enter it, causing an internal avalanche of infections/activations (see Kegg Metabolic in Fig 7 at *f*_*o*_ = 40%).

**Fig 9:**
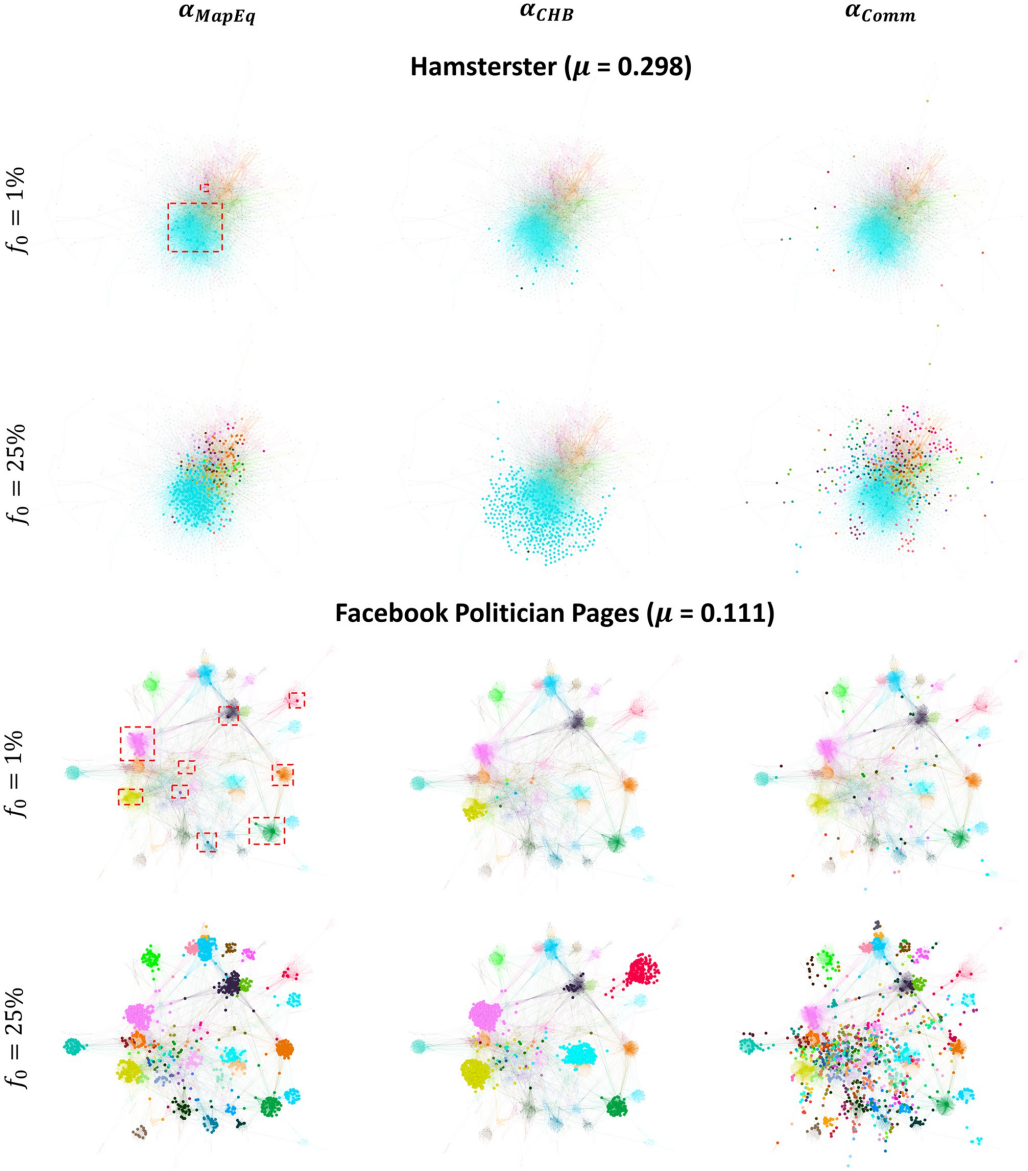
Comparing the position of the top nodes in the Hamsterster and Facebook Politician networks. The top nodes are chosen at a low budget availability (*f*_*o*_ = 1%) and medium budget availability (*f*_*o*_ = 25%). The bigger nodes in the left, middle, and right figures are the top nodes ranked by Map Equation Centrality (*α*_*MapEq*_), Community Hub-Bridge (*α*_*CHB*_), and Comm Centrality (*α*_*Comm*_), respectively.

**(3) Why is it more beneficial to target hub-like nodes at low budget availability in the LT model?** Results reveal that the dynamics on the LT model contrast with that of the SI, SIR, and IC models. Indeed, bridge-like nodes are always preferred in the latter set of models when the budget is limited. However, with the LT model, under limited budget availability, hub-like nodes targeted by the Map Equation Centrality (*α*_*MapEq*_) diffuse better the virus/information across the network. To understand why this phenomenon occurs, we visualize two structurally different networks, namely the Hamsterster and Facebook Politician Pages networks in Fig 9. In these two networks, the Map Equation Centrality (*α*_*MapEq*_) shows good performances up to a specific budget. Suppose that a piece of information is circulating around a given community. If this community is well-connected (i.e., if it has a high internal density compared to its external connections), the piece of information will never enter it [40, 41]. This trend is even more pronounced when the nodes have a high threshold, even if the network has a weaker community structure.

The Map Equation Centrality (*α*_*MapEq*_) overcomes this obstacle in the LT model by targeting nodes inside all the network communities and not around them. Because the Map Equation Centrality (*α*_*MapEq*_) correlates with the node’s intra-community links, the random walker has a higher chance of staying in nodes with a high internal degree. Thus, these nodes tend to be hub-like rather than bridge-like nodes, as seen in the two networks in Fig 9. For demonstration purposes, the nodes targeted by Community Hub-Bridge (*α*_*CHB*_) and Comm Centrality (*α*_*Comm*_) are also shown. These two measures perform poorly on the LT model when the budget is limited.

As we can see, Community Hub-Bridge (*α*_*CHB*_) picks many nodes inside a few communities, missing many regions in the network. Concerning Comm Centrality (*α*_*Comm*_), since the top nodes picked are more frequent between the communities rather than the inside as it has a preference for bridge-like nodes, this will not be enough at low budget availability for the piece of information to enter the tightly-knit communities given the complex contagion dynamics of the LT model [40, 41]. Therefore, at a low budget availability, ensuring a piece of information starts by nodes embedded in their communities such that these communities spread across all the network regions is the best approach for effective diffusion. If bridge-like nodes are targeted at low budget availability, the information will not be capable of entering high-density communities. Note that this behavior contrasts with the behaviors seen with the SI, SIR, and IC models. Indeed, in the latter set of models and at low budget availability, bridge-like nodes play the most influential role in diffusion since, in these models which follow the simple contagion dynamics, bridge-like nodes have a higher chance to enter many communities and cause an avalanche of activations/infections.

To go further, Centola and Macy [49] have theoretically proven that complex contagions, such as contagions that spread in the LT model, differ from simple contagions, by tackling Granovetter’s seminal work on the “strength of weak ties” [50]. They showed that not all contagions spread the same way on weak ties, ties that connect different communities. For simple contagions, it suffices one single contact such as the spread of disease or information. However, complex contagions need social reinforcement, as they involve costly or risky actions. If bridges are not wide, that is they only bridge two distant clusters but do not have several connections in the community they belong to, complex contagions cannot be easily diffused as the probability of social reinforcement taking place is very low. These results go hand in hand with the study presented in this article. Community-aware centrality measures are divided into two main groups: those prioritizing hubs, and those targeting bridges that connect distant communities. As demonstrated in the LT model which depicts the diffusion of complex contagions, to achieve higher activation on low budget availability, diffusion must start from within the communities. This is why as Map Equation Centrality outperforms the other measures. Indeed, we can clearly see that the bridge-like nodes selected lack extensive connections within their communities, such as the ones chosen by Comm Centrality. This renders the diffusion of a complex contagion challenging and, and at low budget availability, impossible, as social reinforcement through those bridges is unattainable.

**(4) Why is it more beneficial to target both hub-like and bridge-like nodes simultaneously or bridge-like nodes only at high budget availability in the LT model?**

**Fig 10:**
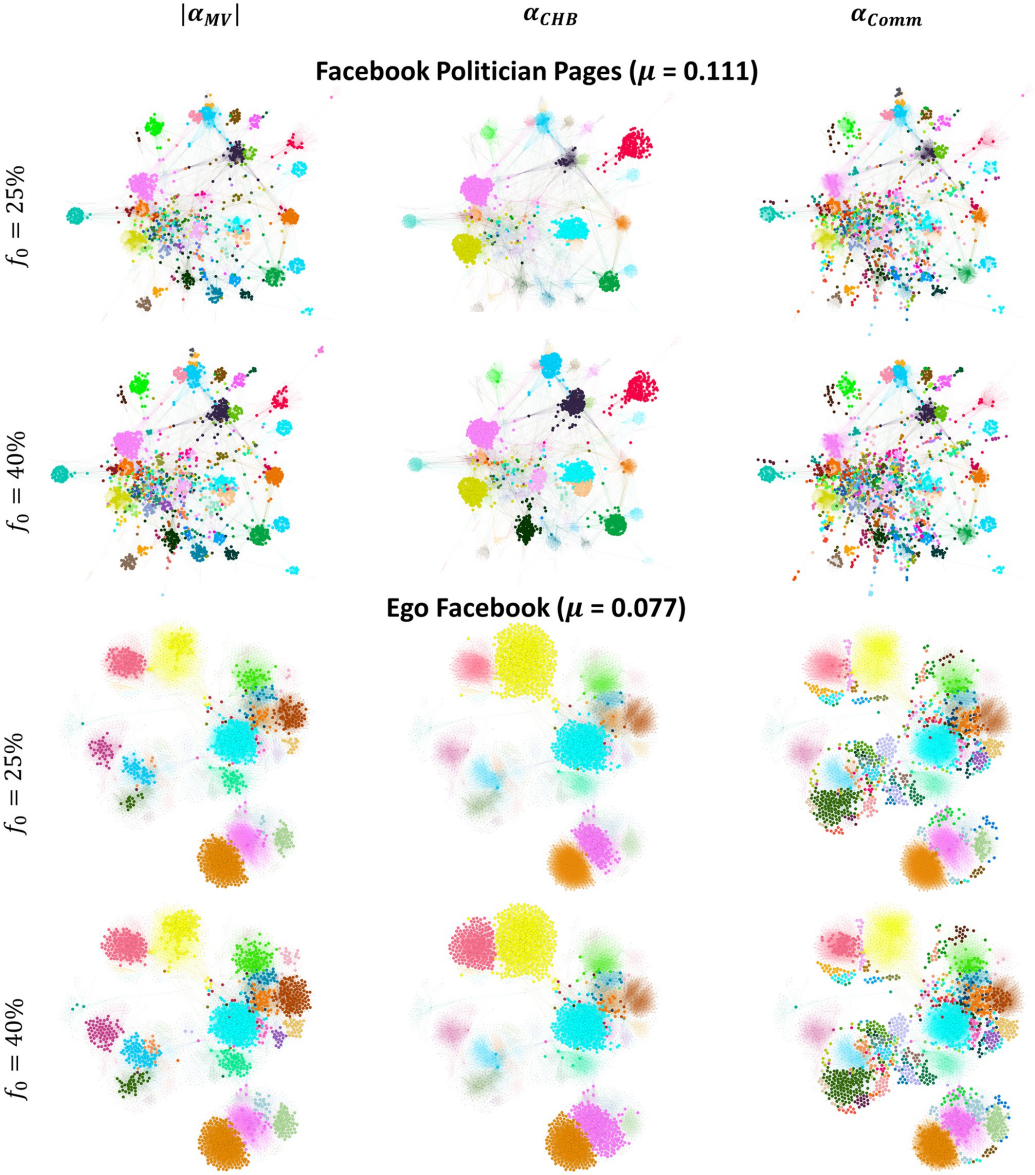
Comparing the position of the top nodes in the Facebook Politician Pages and Ego Facebook networks. The top nodes are chosen at a medium budget availability (*f*_*o*_ = 25%) and high budget availability (*f*_*o*_ = 40%). The bigger nodes in the left, middle, and right figures are the top nodes ranked by Modularity Vitality targeting hubs and bridges (|*α*_*MV*_|), Community Hub-Bridge (*α*_*CHB*_), and Comm Centrality (*α*_*Comm*_), respectively.

At high budget availability, results show that either hub-like and bridge-like nodes targeted simultaneously using Modularity Vitality targeting hubs and bridges (|*α*_*MV*_|) or bridge-like nodes only using Comm Centrality (*α*_*Comm*_) yield the highest outbreak. These trends also contrast with the ones found in the SI, SIR, and IC models. At high budget availability, the networks with the latter set of models showed good performance with either hub-like nodes targeted with Modularity Vitality targeting hubs ($\alpha_{MV}^{+}$) or hub-like nodes targeted with Modularity Vitality targeting bridges ($\alpha_{MV}^{-}$), depending on the community structure strength. This leads us to investigate why the LT dynamics also diverge when the budget availability is high. We visualize Ego Facebook and Facebook Politician Pages to depict the two trends regarding the outperformance of Modularity Vitality targeting hubs and bridges (|*α*_*MV*_|) in the former network and bridge-like nodes only using Comm Centrality (*α*_*Comm*_) in the latter network in Fig 10. For comparison purposes, we also choose Community Hub-Bridge (*α*_*CHB*_) to be represented as well.

Discussing first the Facebook Politician Pages network, we can see that both Modularity Vitality targeting hubs and bridges (|*α*_*MV*_|) and Comm Centrality (*α*_*Comm*_) target nodes that all well-distributed, internally and externally, across all the communities in the network, unlike Community Hub-Bridge (*α*_*CHB*_) which targets a limited number of communities. Since the communities in Facebook Politician Pages are not of equivalent sizes, Comm Centrality (*α*_*Comm*_) yields a higher activation rate as the difference between Modularity Vitality targeting hubs and bridges (|*α*_*MV*_|) and Comm Centrality (*α*_*Comm*_) is that the latter targets more nodes on the peripherical communities. In contrast, in the Ego Facebook network, since there is a smaller variance in the community size distribution, targeting hub-like and bridge-like nodes simultaneously using Modularity Vitality targeting hubs and bridges (|*α*_*MV*_|) assures that the diffusion will spread across the communities as small peripherical communities do not exist.

**Fig 11:**
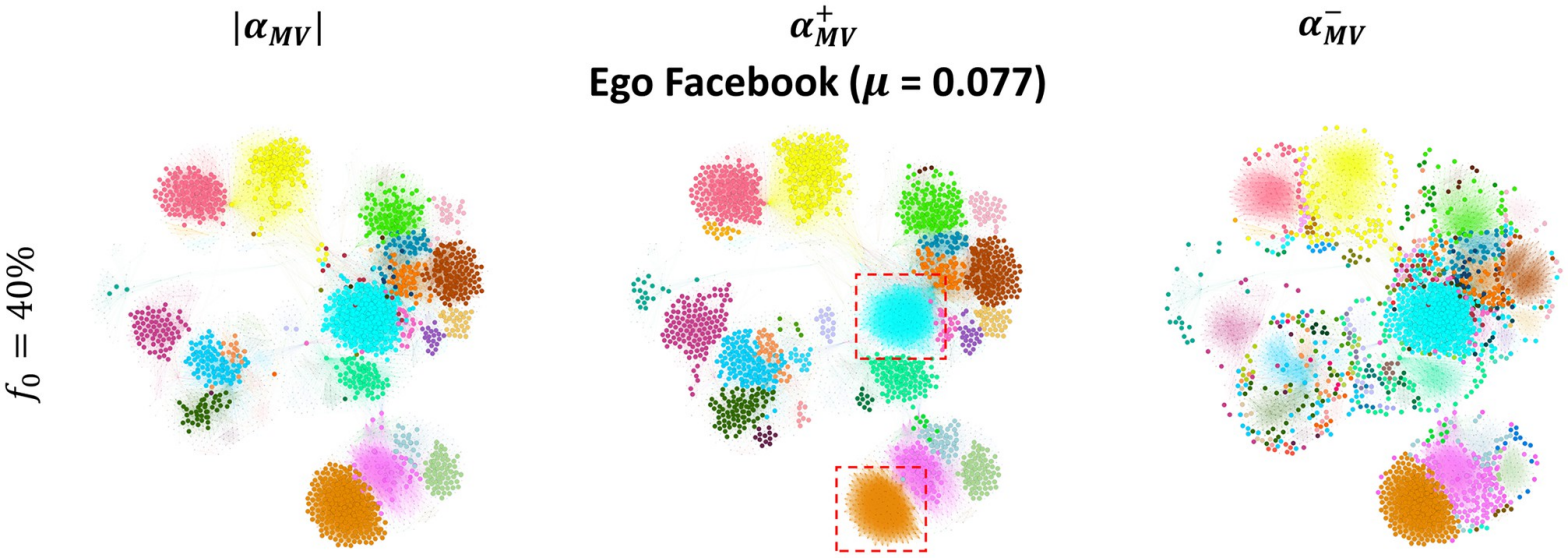
Comparing the position of the top nodes in the Ego Facebook networks. The top nodes are chosen at a high budget availability (*f*_*o*_ = 40%). The bigger nodes in the left, middle, and right figures are the top nodes ranked by Modularity Vitality targeting hubs and bridges (|*α*_*MV*_|), hubs only ($\alpha_{MV}^{+}$), and bridges only ($\alpha_{MV}^{-}$), respectively.

The question is, why do we observe such behavior in the LT model rather than the behavior seen with Modularity Vitality targeting hubs ($\alpha_{MV}^{+}$) and Modularity Vitality targeting bridges ($\alpha_{MV}^{-}$) in the SI, SIR, and IC dynamics. Targeting nodes inside the communities satisfies the complex contagion dynamical conditions of the LT model for a higher activation rate [40, 41]. However, with a higher budget availability, bridge-like nodes also play a role since many are targeted. Subsequently, at high budget availability in the LT dynamics, enough hub-like and bridge-like nodes will make the diffusion spread farther, rather than just targeting hub-like nodes or bridge-like nodes independently. Moreover, as we discussed previously, a major drawback for Modularity Vitality targeting hubs ($\alpha_{MV}^{+}$) is that it misses hub-like nodes in big communities since they are easily replaced by others and subsequently they do not receive a high centrality score. Hence, it falls back in the LT dynamics as all communities should be targeted internally before externally for higher activation rates. We visualize the Ego Facebook network in Fig 11 with the top 40% nodes ranked by all the Modularity Vitality variants (i.e., $\alpha_{MV}^{+}$, $\alpha_{MV}^{-}$, and |*α*_*MV*_|) to show how Modularity Vitality targeting both hubs and bridges (|*α*_*MV*_|) is well suited for the LT dynamics as it assures internal diffusion and external diffusion by effectively utilizing the high budget availability. The red dashed lines highlight that two large communities in the network are not targeted by Modularity Vitality targeting hubs ($\alpha_{MV}^{+}$) despite having a budget of *f*_*o*_ = 40%. In contrast, Modularity Vitality targeting both hubs and bridges (|*α*_*MV*_|) targets hub-like nodes inside all the communities and a set of bridges between them.

The best performing community-aware centrality measures under the different community structure strength, budgets, and models are summarized in Table 2.

**Table 2:** Summary of the best performing community-aware centrality measures.

Community structure strength	Budget	SI	SIR	IC	LT
Strong community structure	Low	*α*_*Comm*_ (bridge-like)	*α*_*Comm*_ (bridge-like)	*α*_*Comm*_ (bridge-like)	*α*_*MapEq*_ (hub-like)
Strong community structure	High	$\alpha_{MV}^{-}$ (bridge-like)	$\alpha_{MV}^{-}$ (bridge-like)	$\alpha_{MV}^{-}$ (bridge-like)	|*α*_*MV*_| (hubs & bridges), *α*_*Comm*_ (bridge-like)
Weak community structure	Low	*α*_*Comm*_ (bridge-like)	*α*_*Comm*_ (bridge-like)	*α*_*Comm*_ (bridge-like)	*α*_*MapEq*_ (hub-like)
Weak community structure	High	$\alpha_{MV}^{+}$ (hub-like)	$\alpha_{MV}^{+}$ (hub-like)	$\alpha_{MV}^{+}$ (hub-like)	|*α*_*MV*_| (hubs & bridges), *α*_*Comm*_ (bridge-like)

**(5) Comparing the dynamics of communities identified by Infomap and Louvain, why does the behavior of the community-aware centrality measures in the SI, SIR, and IC diverge when the budget is low to medium?**

**Fig 12:**
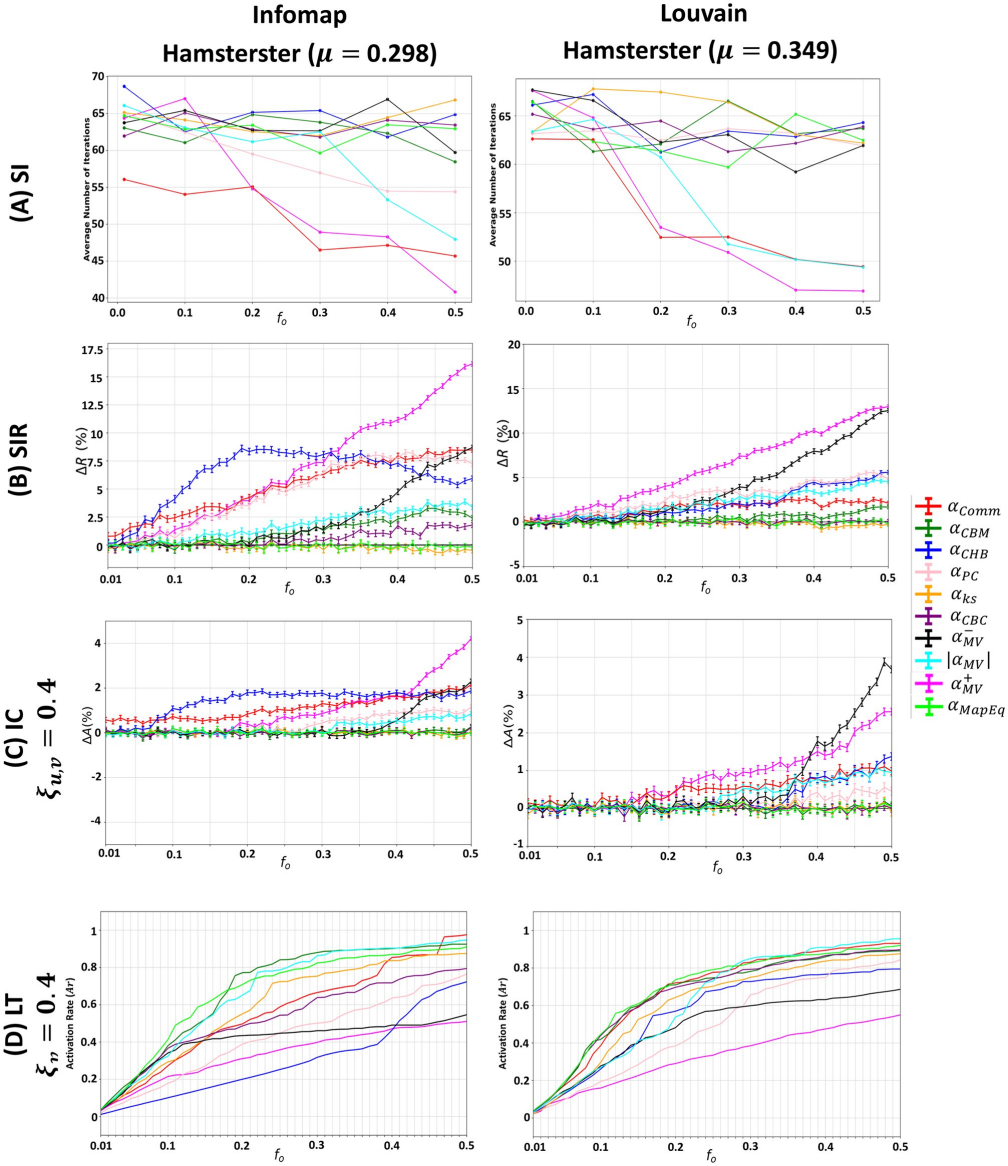
Comparing the trends of the various dynamic models in Hamsterster with its communities identified by Infomap and Louvain. The first, second, third, and fourth rows indicate the results of the (A) SI model, (B) SIR model, (C) IC model, (D) LT model.

Results show that in the SI, SIR, and IC models, with a budget availability varying from low to medium, the nodes targeted by the community-aware centrality measures induce different dynamics when Infomap identifies the communities compared to the communities identified by Louvain. The measures having preferences for bridge-like nodes, namely Comm Centrality (*α*_*Comm*_) and Community Hub-Bridge (*α*_*CHB*_), underperform with Louvain as shown in the first three rows of Fig 12 for the Hamsterster network.

**Fig 13:**
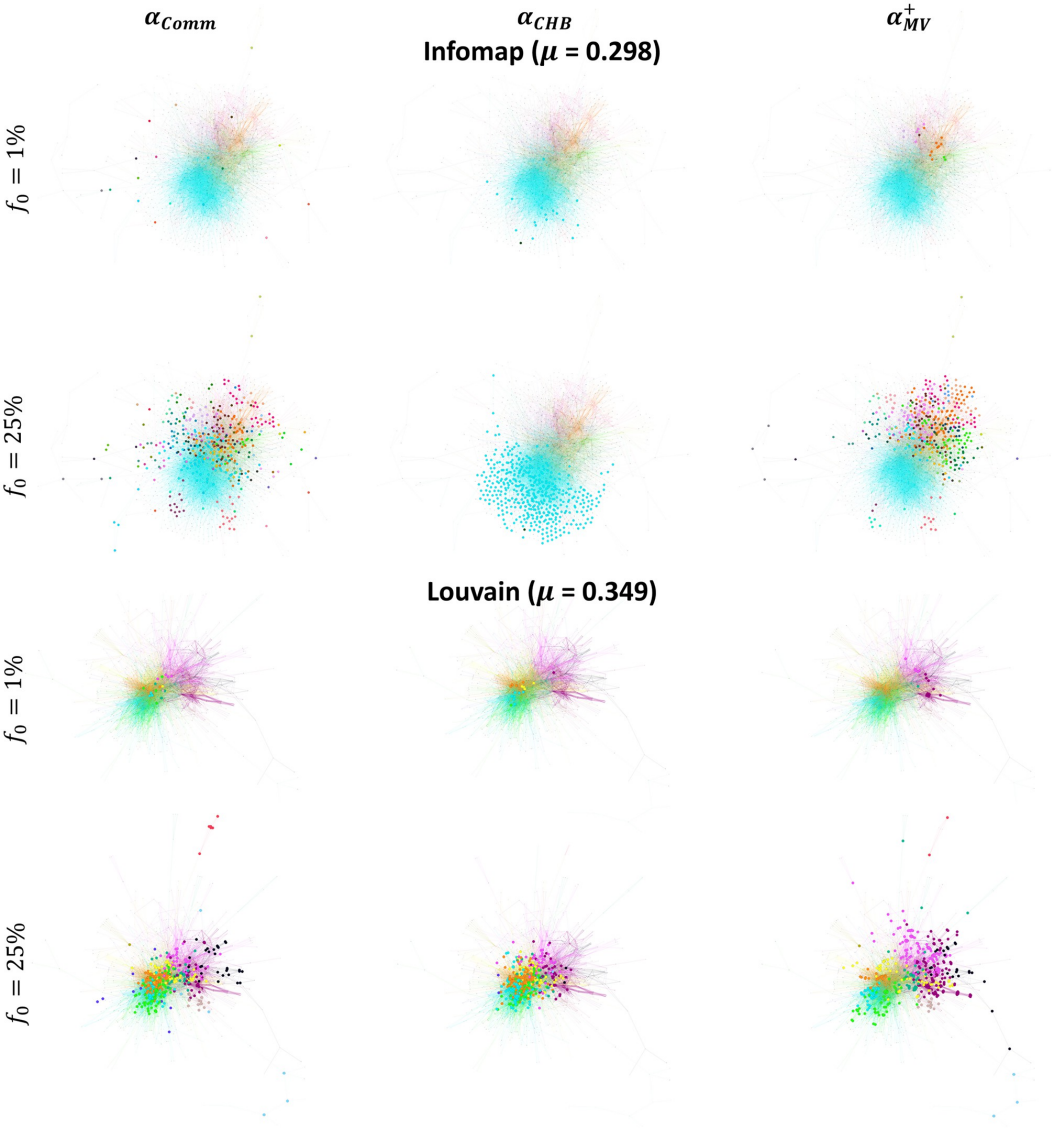
Comparing the position of the top nodes in the Hamsterster network having its communities identified by Infomap and Louvain. The top nodes are chosen at a low budget availability (*f*_*o*_ = 1%) and medium budget availability (*f*_*o*_ = 25%). The bigger nodes in the left, middle, and right figures are the top nodes ranked by Comm Centrality (*α*_*Comm*_), Community Hub-Bridge (*α*_*CHB*_), and Modularity Vitality targeting hubs ($\alpha_{MV}^{+}$), respectively.

**Fig 14:**
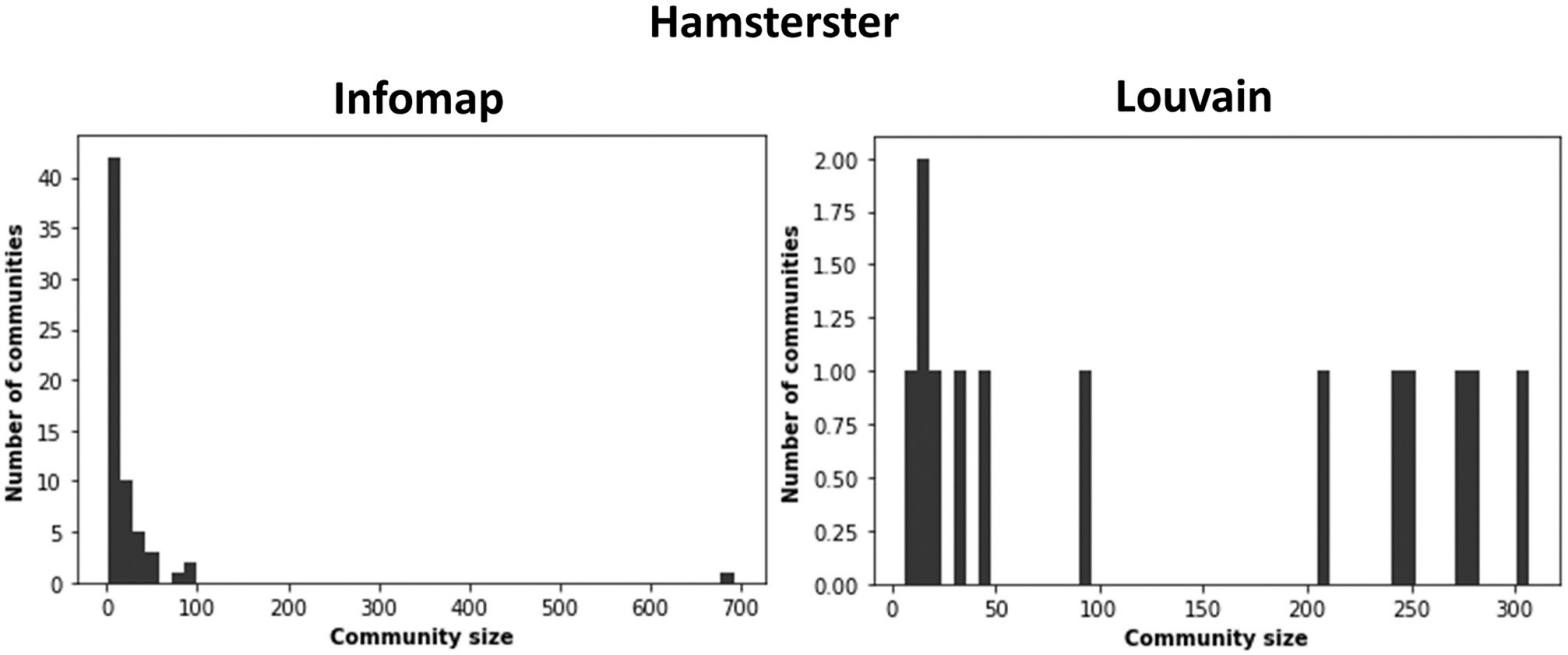
Histograms of the community size distribution of the Hamsterster network. Communities are identified by Infomap and Louvain.

We visualize the Hamsterster network with its communities identified by Infomap and Louvain to clarify why this occurrence happens in Fig 13. We also show the histogram of the community size distribution in Fig 14 of the Hamsterster network using both Infomap and Louvain.

Generally, Infomap yields high variance in the sizes of the communities with a power-law distribution. Louvain uncovers fewer communities with more uniform sizes having a lower variance. For instance, in the Hamsterster network, Infomap identifies 64 communities with a maximum size of 692 and a minimum size of 2. Conversely, Louvain uncovers 13 communities with a maximum size of 307 and a minimum size of 6. As Comm Centrality (*α*_*Comm*_) exploits bridge-like nodes in all of the communities of the network, either small or large, having a more uniform size distribution with less variance diminishes Comm Centrality’s power. Indeed, the bridge-like nodes’ frequency undoubtedly decreases with Louvain. As we can see in Fig 13, when the budget is *f*_*o*_ = 1%, and the communities are identified by Infomap, Comm Centrality’s top nodes are well-distributed across the network in opposition to its top nodes selected when communities are determined by Louvain where they are situated in the core of the network. Similarly is the case at *f*_*o*_ = 25%.

**(6) Comparing the dynamics on communities identified by Infomap and Louvain, why do the dynamics of the community-aware centrality measures in the LT diverge when the budget availability is high?**

Results show that the dynamics of the LT model differ from that of the SI, SIR, and IC even when uncovering the communities with the Louvain algorithm. As the findings generally show, the differences in the outbreak can be seen when the budget is limited with the stated set of models. However, with the LT model, the differences are featured at high budget availability (see the last row of Fig 12). These differences accentuate how the LT dynamics differ from the remaining models. Yet, results show that bridge-like nodes also play a lesser role when Louvain identifies the communities. This is clear when *f*_*o*_ exceeds 0.47 in the LT dynamics of Hamsterster in the last row of Fig 12. At this given budget range, Comm Centrality (*α*_*Comm*_) outperformed the remaining measures with Infomap. However, Modularity Vitality targeting hubs and bridges (|*α*_*MV*_|) outperformed with Louvain. In addition, we note that Map Equation Centrality (*α*_*MapEq*_) and Community-based Centrality (*α*_*CBC*_) show superior performances with Louvain as these measures prioritize hub-like nodes, which are pervasive with Louvain as compared to Infomap.

## 7 Conclusion

Modeling complex network dynamics is a major breakthrough in describing and understanding the real world. Researchers from various disciplines, such as sociology, epidemiology, and physics, have developed diffusion models deemed to be interdisciplinary in nature. These diffusion models differ mainly in their underlying conditions and states as the dynamic process begins in a given network. In the vast data era we live in, a myriad of unique topological characteristics characterizes networks. One of the prominent characteristics is the network’s community structure. Indeed, the community structure affects any diffusive phenomena on the network.

That being said, finding the most important nodes that play a role in accelerating or inhibiting a diffusion phenomenon within and across these communities is of utmost importance. Community-aware centrality measures acknowledge the network’s community structure and aim to identify key nodes accordingly. Some measures prioritize hub-like nodes, while others prioritize bridge-like nodes. Still, the aim at the end is to maximize the diffusion (or inhibit it) under any dynamic model in a network.

Numerous community-aware centrality measures and diffusion models have been proposed in the literature. This article investigates the interplay between the diffusion dynamics, the community-aware centrality measures, and the network’s topological characteristics. More specifically, we analyze how the diffusive power of nodes selected based on various community-aware centrality measures changes with respect to the model and the network at hand. Four diffusion models have been simulated, starting with a set of initial nodes based on the community-aware measures under study on synthetic and real-world networks. The diffusion models studied are the Susceptible-Infected (SI), Susceptible-Infected-Recovered (SIR), Independent Cascade (IC), and Linear Threshold (LT) models.

Results show that the strength of the community structure and budget availability significantly impact how diffusion spreads. Furthermore, the SI, SIR, and IC dynamics, which belong to simple contagions, show a convergent behavior, while the LT dynamics, belonging to complex contagions, diverge within a given community structure strength and budget availability. By controlling the community structure strength in synthetic networks, we observed that the community-aware centrality measures are more profitable in networks with a strong community structure strength. With real-world networks with a strong community structure under the SI, SIR, and IC dynamics, bridges are always preferred regardless of the budget. With the LT dynamics, hub-like nodes are preferred when the budget is limited or high. However, when the budget increases, hub-like and bridge-like nodes are preferred. In networks with a weak community structure, with the SI, SIR, and IC dynamics, bridge-like nodes are preferred, then distant hub-like nodes take over at high budget availability. However, with the LT dynamics, hub-like nodes are preferred at a low budget, while more interlinked nodes with hub-like nodes are preferred from medium to high budget availability. We also analyzed the impact of the community detection algorithm, and results showed that in the SI, SIR, and IC dynamics, the performance of the measures changes when the budget is limited. In contrast, with the LT dynamics, differences are seen when the budget availability is high. The differences between the diffusion models, mainly seen at a limited budget availability, is credited to the fact that the conditions in the SI, SIR, and IC models are well suited to select bridge-like nodes as it is easier for the virus/piece of information to circulate from one community to another compared to the LT model. Indeed, if the virus/piece of information is initiated in the well-connected communities under the LT dynamics, the community will never be part of the occurring diffusive phenomenon.

The extensive experiments shed light on how the diffusion dynamics, the position of the nodes initially activated, the network’s community structure strength, and the budget availability are interconnected. Given the knowledge of one or the other, one can choose the suitable measure for running the most effective diffusion on the network.

## Supporting information

S1 Text Supplementary material. (DOCX)
