# Correction: On the role of diffusion dynamics on community-aware centrality measures

**DOI:** 10.1371/journal.pone.0321504

**Published:** 2025-04-01

**Authors:** Stephany Rajeh, Hocine Cherifi

There is an error in Table 1, where the first row should not be divided into two rows but merged into a single row (column headings). Please see the correct Table 1 here.

**Table 1:** A summary of the studies of community-aware centrality measures. SIR means Susceptible-Infected-Recovered model and LT refers to Linear Threshold model. The character ‘-’ refers to “not applicable”.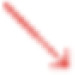 indicates that the goal is to minimize diffusion and 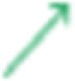 indicates that the goal is to maximize diffusion.

Community-aware centrality measures	Diffusion model & goal	Node selection method	Real-world networks	Synthetic networks	Number of community detection algorithms
Participation Coefficient [4]	–	–	12	–	1
Community-based Centrality [5]	SIR 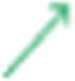	Single	6	–	5
Comm Centrality [6]	SIR 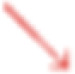	Multiple	4	3	1
K-shell with Community [7]	SIR 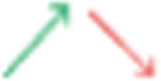	Single & Multiple	4	–	1
Community-based Mediator [8]	SIR 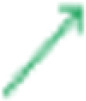	Single & Multiple	5	2	1
Community Hub-Bridge [9]	SIR 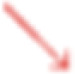	Multiple	6	5	3
Modularity Vitality [10]	SIR 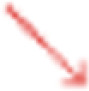	Multiple	2	3	1
Map Equation Centrality [11]	SIR 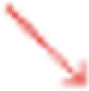 & LT 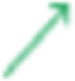	Single & Multiple	12	1	2
