# Supplementary material for: On the role of diffusion dynamics on community-aware centrality measures
Source: PLoS One. 2024 Jul 18;19(7):e0306561. doi: 10.1371/journal.pone.0306561 (PMC11257236; doi:10.1371/journal.pone.0306561)
Supplement: S1 Text — (DOCX) [file pone.0306561.s001.docx]

**On the role of diffusion dynamics on community-aware centrality measures**

Stephany Rajeh^1,2*^, Hocine Cherifi^3^

^1^ Efrei Research Lab, EFREI Paris-Pantheon-Assas University, Villejuif, France

^2^ LIP6 CNRS, Sorbonne University, Paris, France

^3^ ICB UMR 6303 CNRS, University of Burgundy, Dijon, France

*Corresponding Author

Email: stephany.rajeh@efrei.fr

**Supplementary Materials and Methods**

## Community-aware Centrality Measures

Let $G(V,E)$ be an undirected and unweighted graph where $V$ is the set of nodes, $E⊑V\times V$ is the set of edges, and $N=|V|$ is the total size of the network. The connections between the nodes are represented in the adjacency matrix $A=(a_{i,j})$ such that $a_{i,j}=1$, if node $i$ is connected to node $j$ and $a_{i,j}=0$, otherwise. Let graph $G$ is divided to $C=\{c_{1},c_{2},\ldots, c_{q}, \ldots, c_{|C|}\}$ communities where $c_{q}$ is $q$-th community, $|C|$ is the total number of communities, and $n_{c_{q}}$ is the total number of nodes in community $c_{q}$. In a non-overlapping community structure, a node $i$ is a member of a single community $c_{q}$, therefore $c_{q}\cap c_{l}=\emptyset\forall q\neq l$. Intra-community edges link nodes in the same community, while inter-community edges join nodes in different communities. More formally, $\left| E_{c_{q}}^{in} \right|=\frac{1}{2}\sum_{i,j\in c_{q}} A_{i,j}$ and $\left| E_{c_{q}}^{out} \right|=\frac{1}{2}\sum_{i\in c_{q}} \sum_{j\in C\backslash c_{q}} A_{i,j}$ denote, respectively, the number of intra-community and inter-community edges of community $c_{q}$.

A node $i$ has a total degree of $k_{i}=\sum_{j=1}^{N} A_{i,j}=k_{i}^{intra}+k_{i}^{inter}$ where $k_{i}^{intra}$ is the internal degree and $k_{i}^{inter}$ is the external degree. More formally, $k_{i}^{intra}=\sum_{j=1}^{N} A_{i,j}\delta(c_{i},c_{j})$ and $k_{i}^{inter}=\sum_{j=1}^{N} A_{i,j}(1-\delta(c_{i},c_{j}))$ where $\delta(c_{i},c_{j})$ is the Kronecker delta function, indicating that $\delta\left( m,n \right)=1$ if $m=n$, otherwise $\delta\left( m,n \right)=0$, $c_{i}$ denotes community of node $i$, and $c_{j}$ denotes the community of node $j$. Moreover, a node $i$ has a degree in community $c_{q}$ denoted as $k_{i,c_{q}}$. In other words, $k_{i,c_{q}}$ is the number of links node $i$ has, reaching community $c_{q}$, defined as $k_{i,c_{q}}=\sum_{j=1}^{N} A_{i,j}\delta(c_{i},c_{q})$. It is important to understand that the distinction between $k_{i}^{intra}$ and $k_{i,c_{q}}$ lies in the fact that the former represents the overall internal degree of the node across all communities, while the latter refers to the internal degree of the node within a particular community $c_{q}$.

### 1. Participation Coefficient

Participation Coefficient [1] quantifies the node's importance based on its participation in various communities through its inter-community links. The more diversified across the communities a node's links are, the higher its Participation Coefficient. If the node has only intra-community links, its Participation Coefficient reduces to zero. It is defined as follows:

$$\alpha_{PC}\left( i \right)=1-\sum_{q=1}^{|C|} \left( \frac{k_{i,c_{q}}}{k_{i}} \right)^{2}$$

### 2. Community-based Centrality

Community-based Centrality [2] places importance on the distribution of a node's links in its community and across the other communities. The size of the communities it is connected to is also part of the measure. Indeed, the community size either undermines or enhances the node's influence. It is defined as follows:

$$\alpha_{CBC}\left( i \right)=\sum_{q=1}^{|C|} k_{i,c_{q}}\left( \frac{n_{c_{q}}}{N} \right)$$

### 3. Comm Centrality

Comm Centrality [3] differentiates hubs (high-degree nodes) from bridges (the link between communities) based on a weighted combination of the intra-community links and inter-community links while giving bridges a higher priority. It is defined as follows:

$$\alpha_{Comm}\left( i \right)=\left( 1+\mu_{c_{q}} \right)\times\left( \frac{k_{i}^{intra}}{\max\left( j\in c_{q} \right)k_{i}^{intra}}\times R \right)+\left( 1-\mu_{c_{q}} \right)\times\left( \frac{k_{i}^{inter}}{\max\left( j\in c_{q} \right)k_{i}^{inter}}\times R \right)^{2}$$

where $\mu_{c_{q}}$ is the fraction of inter-community links over the total community links in the community, and $R$ is a user-defined value to standardize the intra-community and inter-community values.

### 4. K-shell with Community

K-shell with Community [4] identifies hubs and bridges depending on their hierarchical position as determined by their $k$-shell after dividing the network into two components. The first comprises the intra-community links, characterizing the node's local influence. The second comprises the inter-community links, characterizing the node's global influence. Then a weighted linear combination of the two influences is computed to assess the node's importance. It is defined as follows:

$$\alpha_{ks}\left( i \right)=\delta\times\alpha^{intra}\left( i \right)+(1-\delta)\times\alpha^{inter}(i)$$

where $\alpha^{intra}\left( i \right)$ and $\alpha^{inter}\left( i \right)$ refer to the $k$-shell value of node $i$ on the graphs constituting intra-community links and inter-community links, respectively. In this study, $\delta$ is equal to 0.5 so neither hubs nor bridges are preferentially selected.

### 5. Community-based Mediator

Community‑based Mediator [5] identifies influential nodes that can quickly spread information across communities based on the entropy of their random walks. The more a node connects communities, the higher its entropy and its importance under the Community‑based Mediator. It is defined as follows:

$$\alpha_{CBM}\left( i \right)=H_{i}\times\frac{k_{i}}{\sum_{i=1}^{N} k_{i}}$$

where $H_{i}=\left[ -\sum\rho_{i}^{intra}log\left( \rho_{i}^{intra} \right) \right]+\left[ -\sum\rho_{i}^{inter}log\left( \rho_{i}^{inter} \right) \right]$ node $i$'s entropy according to $\rho_{i}^{intra}$ and $\rho_{i}^{inter}$ which represent the intra-community and inter-community links over the total degree of node $i$ and $\sum_{i=1}^{N} k_{i}$ represents the sum of the degrees of all the nodes.

### 6. Community Hub-Bridge

Community Hub-Bridge [6] weighs the node's local influence through its intra-community links by the size of the node's belonging community and the node's global influence by the number of neighboring communities a node can reach in one hop. Then, it sums both influences to assess the overall influence. It is defined as follows:

$$\alpha_{CHB}\left( i \right)=n_{c_{q},i}\times k_{i}^{intra}+\sum_{c_{l}\subset C\backslash c_{q}}^{N} \bigvee_{j\in c_{l}} a_{i,j}\times k_{i}^{inter}$$

where $n_{c_{q},i}$ is the size of the community $c_{q}$ node $i$ belongs to and $\bigvee_{j\in c_{l}} a_{i,j}=1$ if node $i$ connects to at least one node $j$ in community $c_{l}$.

### 7. Modularity Vitality

Modularity Vitality [7] identifies hubs and bridges based on their contribution to the network's modularity. One quantifies their contribution through the vitality principle that measures the effect of node removal on a quality measure. Removing hubs tends to decrease the network's modularity, while removing bridges tends to increase it. It is defined as follows:

$$\alpha_{MV}\left( i \right)=Q\left( G \right)-Q(G\{i\})$$

where $Q(G)$ is the network's modularity and $Q(G\{i\})$ is the network's modularity after the removal of node $i$. Note that since Modularity Vitality is a signed community-aware centrality measure, we investigate it using hubs-first $\left( \alpha_{MV}^{+}\left( i \right) \right)$, bridges-first $\left( \alpha_{MV}^{-}\left( i \right) \right)$, and hubs-and-bridges $\left( {| \alpha}_{MV}\left( i \right)| \right)$ ranking schemes.

### 8. Map Equation Centrality

Map Equation Centrality [8] measures the importance of a node in a network by considering the collective marginal harm it causes to the remaining nodes in terms of codeword length, that is, by how many bits the codeword lengths for the remaining nodes could be reduced if the node was silenced. Silencing a node means that when a random walker visits it, the sender does not communicate the codeword for visiting the node to the receiver, resulting in a compressed network modular description. The more one can compress the network's modular description without encoding the node, the higher the node's influence. This means that nodes frequently visited by the random walker play an important role in the network's modular structure. It is defined as follows:

$$\alpha_{MapEq}\left( i \right)=L^{i}-L^{i*}$$

where $L^{i}$ denotes the inefficient code (i.e., the difference in the code length between the coding scheme that assigns codewords to all nodes but does not use node $i$'s codeword) and $L^{i*}$ denotes the efficient code (i.e., the coding scheme that assigns codewords to all nodes but never for node $i$).

## Generation of Synthetic Networks

A set of synthetic networks are generated by the Lancichinetti, Fortunato, and Radicchi (LFR) algorithm [9]. The community structure strength ($\mu$), the degree distribution power-law's exponent ($\gamma$) and the community size distribution power-law's exponent ($\theta$) are varied while keeping the remaining parameters fixed. The values of the parameters set are given in Table 2.

The community structure strength ($\mu$) is tuned according to the fraction of the inter-community links which ranges from 0 to 1. Small values, designating few links between communities, generate networks with a strong community structure while large values, designating many links between communities, generate networks with a loose community structure. To cover three typical cases with respect to the community structure strengths, three networks with a strong ($\mu$ = 0.05), medium ($\mu$ = 0.20), and weak ($\mu$ = 0.70) community structure strengths are generated, while fixing the remaining parameters (i.e., $\theta$ and $ $\gamma$) under investigation at 2.7.

The exponent of the community size distribution ($\theta$) is set to three values, resulting in three networks generated at $\mu$ = 0.05. When $\theta$ = 2, there is a large variance in the community sizes, with few small communities coexisting with large communities. When $\theta$ = 3, a higher frequency of communities exists but there is a smaller variance in the community size distribution (i.e., communities have equivalent sizes). We consider $\theta$ = 2.7 as the reference case, where it indicates that a variance in the community size distribution still exists but is smaller than the case with $\theta$ = 2 and higher than the case with $\theta$ = 3.

The exponent of the degree distribution ($\gamma$) is set to three values as well, resulting in three networks generated at $\mu$ = 0.05. When $\gamma$ = 2, the network generated is characterized by a hub-and-spoke structure. When $\gamma$ = 3, the network generated has many nodes with a similar number of neighbors while ensuring the community structure is maintained. We consider $\gamma$ = 2.7 as the reference case.

## Epidemic Threshold

In the SIR model, the spread of a disease depends on the recovery rate ($\psi$) and the infection rate ($\lambda$). However, the disease is incapable of spreading if the infection rate is less than the epidemic threshold ($\lambda_{th}$) of the network. In this study, we set the infection rate of the networks slightly larger than their epidemic threshold, which is given by [10]:

$$\lambda_{th}=\frac{<k>}{<k^{2}>- <k>}$$

where $<k>$ and $<k^{2}>$ are the first and second moments of the network's degree distribution. Note that in the SI model, the epidemic threshold of the network amounts to 0 since the disease will certainly spread across all the network in the nonexistence of recovery. Note that to run the models, we use the NDlib library^[[1]](#footnote-1)^.

## Evaluation Measures

### Relative Outbreak Difference

For a given centrality measure, we use the spreading outbreak of the degree centrality as a baseline to compare it with the community-aware centrality measures. Accordingly, the relative difference in the outbreak size is defined as:

$$\Delta R=\frac{R_{c}-R_{b}}{R_{b}}$$

where $R_{c}$ is the outbreak size based on the community-aware ranking scheme of any community-aware centrality measure and $R_{b}$ is the outbreak size based on the degree centrality. If $\Delta R$ is positive, the community-aware centrality measure is more effective than the degree centrality. Otherwise, $\Delta R$ is negative. Note that the reason we use degree centrality as a baseline since many of the community-aware centrality measures are a more sophisticated version of it that take into consideration the community structure. Moreover, the degree centrality measure is a very efficient and interpretable measure one can use. Also note that despite measuring the same nodes in the recovered or active state at the end of the diffusion process, the notation $\Delta R$ is used for the Susceptible-Infected-Recovered model while the notation $\Delta A$ is for the Independent Cascade model.

### Average Number of Iterations

In the Susceptible-Infected model, the nodes at the end of the diffusion process will end up all infected. Accordingly, the number of infected nodes in this case cannot be used as an evaluation criterion. To be able to compare the diffusive power of the community-aware centrality measures, the number of iterations needed to infect 50% of the network is used. The number of iterations (i.e., time) a measure needs to infect 50% of the network, the more effective it is. Since the Susceptible-Infected model is run 100 independent simulations, the number of iterations is averaged over the 100 independent simulations.

## Community Detection Algorithms

In real-world networks, if the community structure is not known a priori, a community detection is needed to uncover it. In this study, we use two fundamentally different community detection algorithms to examine the robustness of the community-aware ranking scheme.

### Infomap

The Infomap algorithm [11] exploits the concept of data compression for a random walker. Since a modular structure characterizes most real-world networks, random walkers tend to stay longer inside communities. Codewords can then be used as prefixes for each community. Also, codewords for each node in each community are assigned (which can be reused across different communities). When the random walker is inside the same community, prefix codes used are minimal. Jumping from one community to another, prefix codes increase. The goal is to build a code that detects communities so that the random walker’s shortest description is acquired.

### Louvain

The Louvain algorithm [12] is based on optimizing the modularity of a network. It consists of two iterative steps. First, communities are uncovered by maximizing local modularity between the nodes. Second, a new network is built. It consists of nodes of the communities found in the previous step. The algorithm keeps running to maximize modularity on the new network until no further gain is possible.

**Supplementary Tables**

Table 1 - Time complexity of the community-aware centrality measures.

| Community-aware centrality measure | Time complexity |
| --- | --- |
| Community Hub-Bridge | $O(N^{2}\vert C\vert)$ |
| Participation Coefficient | $O(N<k>)$ |
| Community-based Mediator | $O(N\vert E\vert<k>)$ |
| Comm Centrality | $O(N\vert C\vert)$ |
| Modularity Vitality | $O(N\vert C\vert+\vert E\vert)$ |
| Community-based Centrality | $O(N<k>)$ |
| K-shell with Community | $O(N\vert E\vert)$ |
| Map Equation Centrality | $O(N\log N)$ |

Where:

- $N$ is the total number of nodes.
- $|E|$ is the total number of edges.
- $<k>$ is the average degree.
- $|C|$ is the total number of communities.

Table 2 - Synthetic networks’ parameters generated by the LFR model.

| Network parameter | Value |
| --- | --- |
| Number of nodes | 2500 |
| Average degree | 8 |
| Maximum degree | 27 |
| Community size distribution exponent ($\theta$) | [2, 2.7, 3] |
| Degree distribution exponent ($\gamma$) | [2, 2.7, 3] |
| Minimum community size | 4 |
| Maximum community size | 250 |
| Mixing parameter $(\mu)$ | [0.05, 0.10, 0.15, 0.20, 0.25, 0.30, 0.35, 0.40, 0.70] |

Table 3 - Topological characteristics of real-world networks based on Infomap. $N$ is the total number of nodes. $|E|$ is the total number of edges. $\mu$ is the mixing parameter. $Q$ is the modularity. $ϰ$ is the average internal degree. $\varphi$ is the average internal distance. $\varphi$ is the average internal density.

| Network ($N;\vert E\vert$) | $\mu$ | $Q$ | $ϰ$ | $\varphi$ | $\psi$ |
| --- | --- | --- | --- | --- | --- |
| EU Airlines (417; 2,953) [13] | 0.07 | 0.11 | 3.97 | 1.49 | 0.60 |
| Ego Facebook (4,039; 88,234) [14] | 0.08 | 0.81 | 14.69 | 1.53 | 0.54 |
| U.S. Airports (500; 2,980) [15] | 0.08 | 0.16 | 2.30 | 1.37 | 0.70 |
| Facebook Friends (329; 1,984) [13] | 0.11 | 0.69 | 5.17 | 1.54 | 0.55 |
| Facebook Pol. (5,908; 41,729) [14] | 0.11 | 0.84 | 5.33 | 1.85 | 0.40 |
| Yeast Collins (1,004; 8,319) [13] | 0.12 | 0.75 | 6.99 | 1.52 | 0.62 |
| Malaria Genes (307; 2,812) [13] | 0.13 | 0.63 | 10.42 | 1.47 | 0.60 |
| NetSci (379; 914) [14] | 0.14 | 0.81 | 3.69 | 1.53 | 0.54 |
| PolBooks (105; 441) [15] | 0.15 | 0.52 | 5.39 | 1.57 | 0.51 |
| Reptiles (496; 984) [14] | 0.15 | 0.81 | 2.77 | 1.85 | 0.44 |
| Marvel Partnerships (181; 224) [13] | 0.15 | 0.81 | 2.01 | 1.86 | 0.41 |
| 911AllWords (13,308; 148,035) [16] | 0.16 | 0.05 | 1.72 | 1.63 | 0.60 |
| U.S. Power Grid (4,941; 6,594) [15] | 0.16 | 0.83 | 2.08 | 2.56 | 0.24 |
| Board of Directors (854; 2,745) [13] | 0.16 | 0.82 | 5.16 | 1.49 | 0.58 |
| PGP (10,680; 24,316) [15] | 0.17 | 0.81 | 2.40 | 1.97 | 0.39 |
| Princeton (6,575; 293,307) [14] | 0.19 | 0.33 | 15.74 | 1.77 | 0.48 |
| London Transport (369; 430) [13] | 0.20 | 0.78 | 1.83 | 2.49 | 0.31 |
| EuroRoad (1,039; 1,305) [14] | 0.20 | 0.79 | 1.94 | 2.70 | 0.25 |
| Internet Top. Cog. (197; 243) [13] | 0.20 | 0.75 | 1.91 | 2.23 | 0.33 |
| DNC Emails (849; 10,384) [13] | 0.20 | 0.42 | 5.70 | 1.40 | 0.63 |
| GrQc (4,158; 13,422) [14] | 0.20 | 0.78 | 3.73 | 1.87 | 0.39 |
| Yeast Protein (1,458; 1,993) [14] | 0.24 | 0.75 | 1.94 | 2.04 | 0.32 |
| E. coli Transcription (329; 456) [18] | 0.25 | 0.69 | 1.69 | 1.66 | 0.47 |
| Game of Thrones (107; 352) [13] | 0.27 | 0.48 | 3.80 | 1.63 | 0.42 |
| Jazz (198; 2,742) [15] | 0.27 | 0.44 | 15.51 | 1.51 | 0.54 |
| Les Misérables (77; 254) [15] | 0.27 | 0.55 | 4.30 | 1.45 | 0.58 |
| Blumenau Drug (75; 181) [13] | 0.28 | 0.69 | 1.69 | 1.66 | 0.47 |
| Retweets Copen. (761; 1,029) [14] | 0.29 | 0.70 | 1.80 | 1.93 | 0.34 |
| Hamsterster (1,788; 12,476) [15] | 0.29 | 0.39 | 3.52 | 1.86 | 0.37 |
| Human Protein (1,788; 12,476) [15] | 0.35 | 0.47 | 2.03 | 2.06 | 0.25 |
| Caltech (762; 16,651) [14] | 0.36 | 0.39 | 13.35 | 1.79 | 0.43 |
| Facebook Org. (5,524; 94,219) [13] | 0.37 | 0.59 | 15.09 | 1.95 | 0.27 |
| Interactome Vidal (5,524; 94,219) [13] | 0.39 | 0.58 | 2.08 | 2.20 | 0.30 |
| AstroPh (5,524; 94,219) [14] | 0.42 | 0.56 | 6.62 | 1.89 | 0.41 |
| Internet AS (6,474; 12,572) [18] | 0.43 | 0.55 | 1.78 | 1.94 | 0.35 |
| DeezerEU (28,281; 92,752) [17] | 0.43 | 0.57 | 2.59 | 2.48 | 0.22 |
| DBLP (12,494; 49,579) [13] | 0.43 | 0.55 | 2.54 | 2.26 | 0.20 |
| Adol. Health (2,539; 10,455) [15] | 0.44 | 0.57 | 2.29 | 0.28 | 0.70 |
| Kegg Metabolic (1,865; 5,769) [13] | 0.47 | 0.44 | 1.77 | 1.79 | 0.53 |
| Bible Nouns (1,707; 9,059) [13] | 0.50 | 0.46 | 4.00 | 1.88 | 0.36 |

**Supplementary Figures**

## Susceptible-Infected (SI) model

The curves herewith represent the centrality measures performance: Comm Centrality ($a_{Comm}$), Community-based Mediator ($a_{CBM}$), Community Hub-Bridge ($a_{CHB}$), Participation Coefficient ($a_{PC}$), K-shell with Community ($a_{ks}$), Community-based Centrality ($a_{CBC}$), Modularity Vitality targeting hubs ($a_{MV}^{+}$), Modularity Vitality targeting bridges ($a_{MV}^{-}$), Modularity Vitality targeting hubs and bridges (${|\alpha}_{MV}|$), and Map Equation Centrality ($a_{MapEq}$).


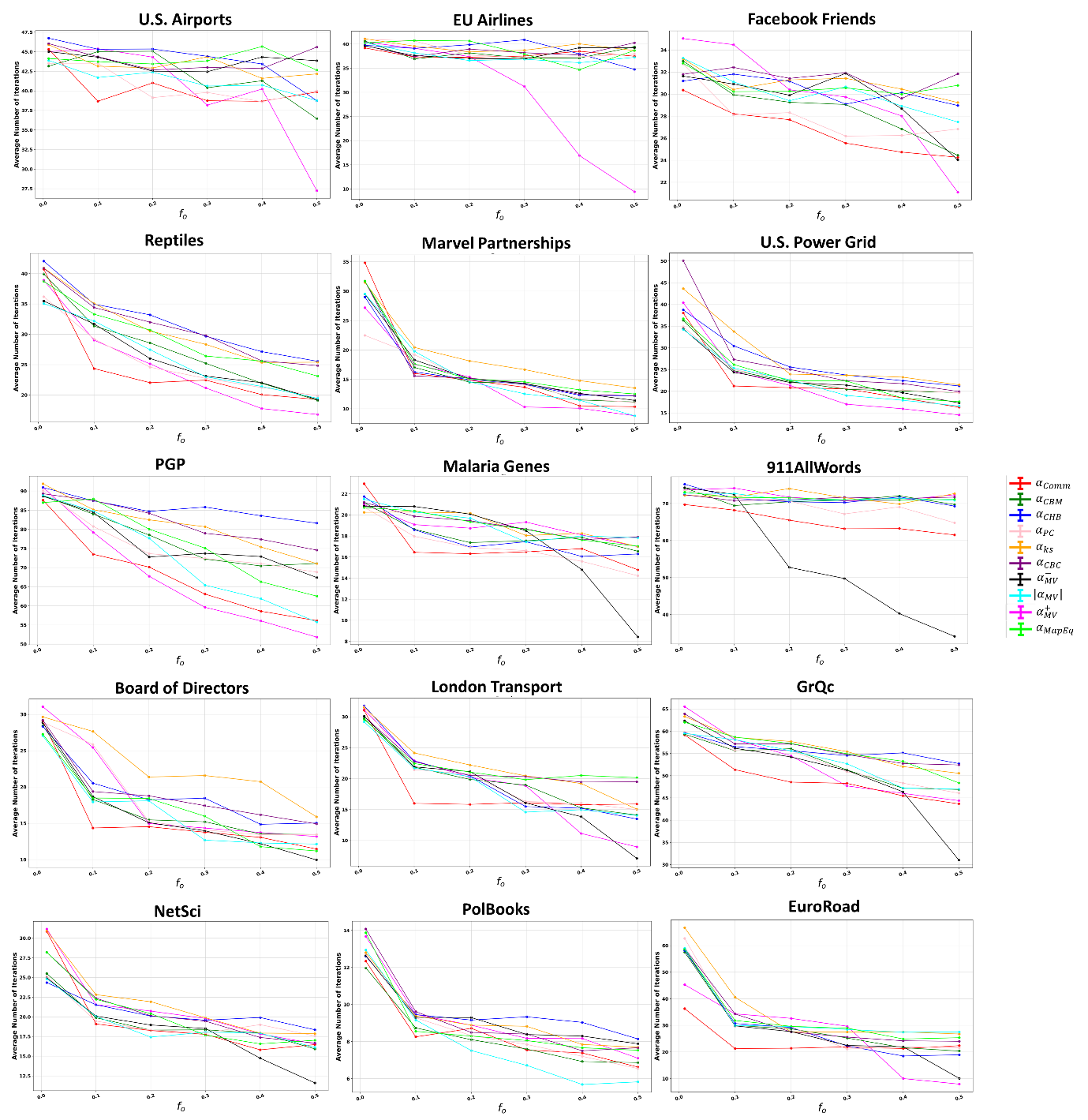


Figure 1 - The trends of the SI model in fifteen real-world networks. The x-axis represents the budget availability or the fraction of initially infected nodes ($f_{o}$) and the y-axis represents the average number of iterations to infect 50% of the network.


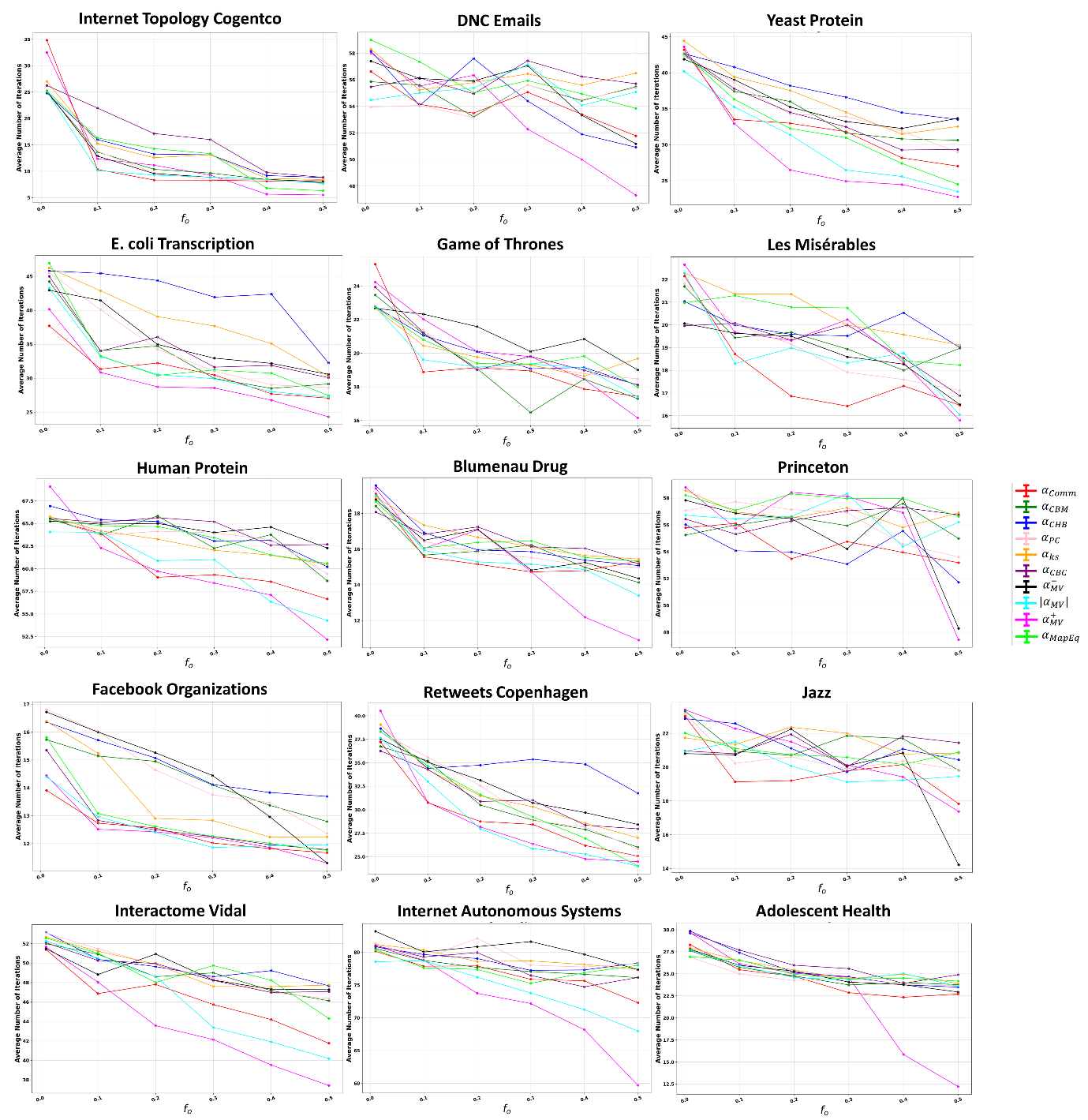


Figure 2 - The trends of the SI model in fifteen real-world networks. The x-axis represents the budget availability or the fraction of initially infected nodes ($f_{o}$) and the y-axis represents the average number of iterations to infect 50% of the network.


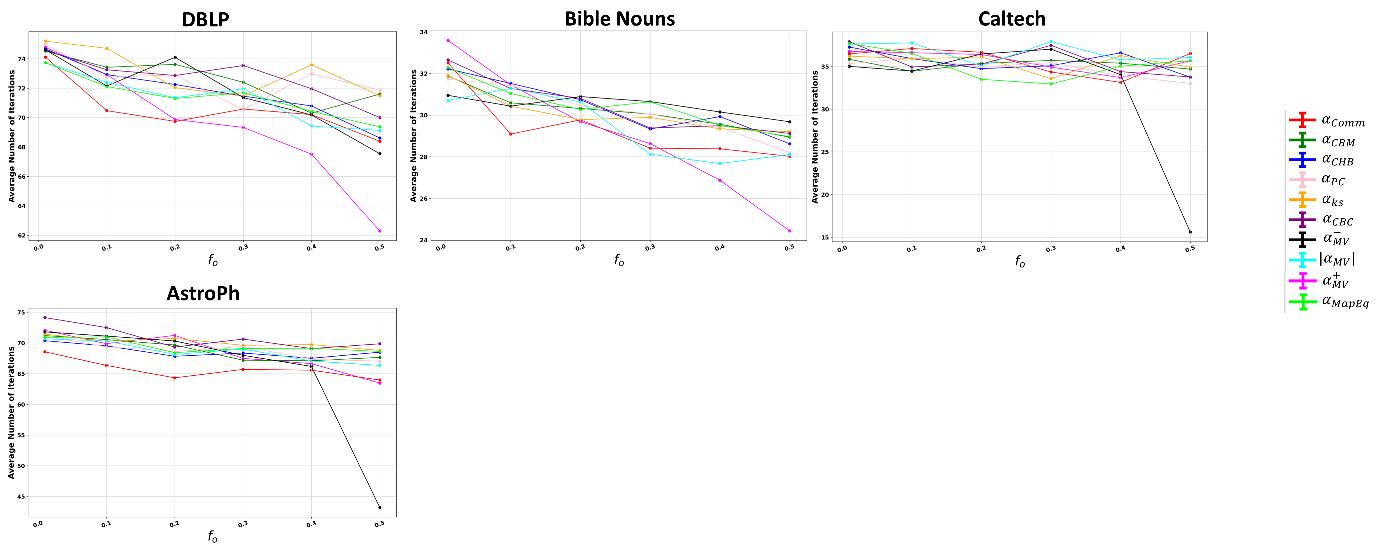


Figure 3 - The trends of the SI model in four real-world networks. The x-axis represents the budget availability or the fraction of initially infected nodes ($f_{o}$) and the y-axis represents the average number of iterations to infect 50% of the network.

## Susceptible-Infected-Recovered (SIR) model


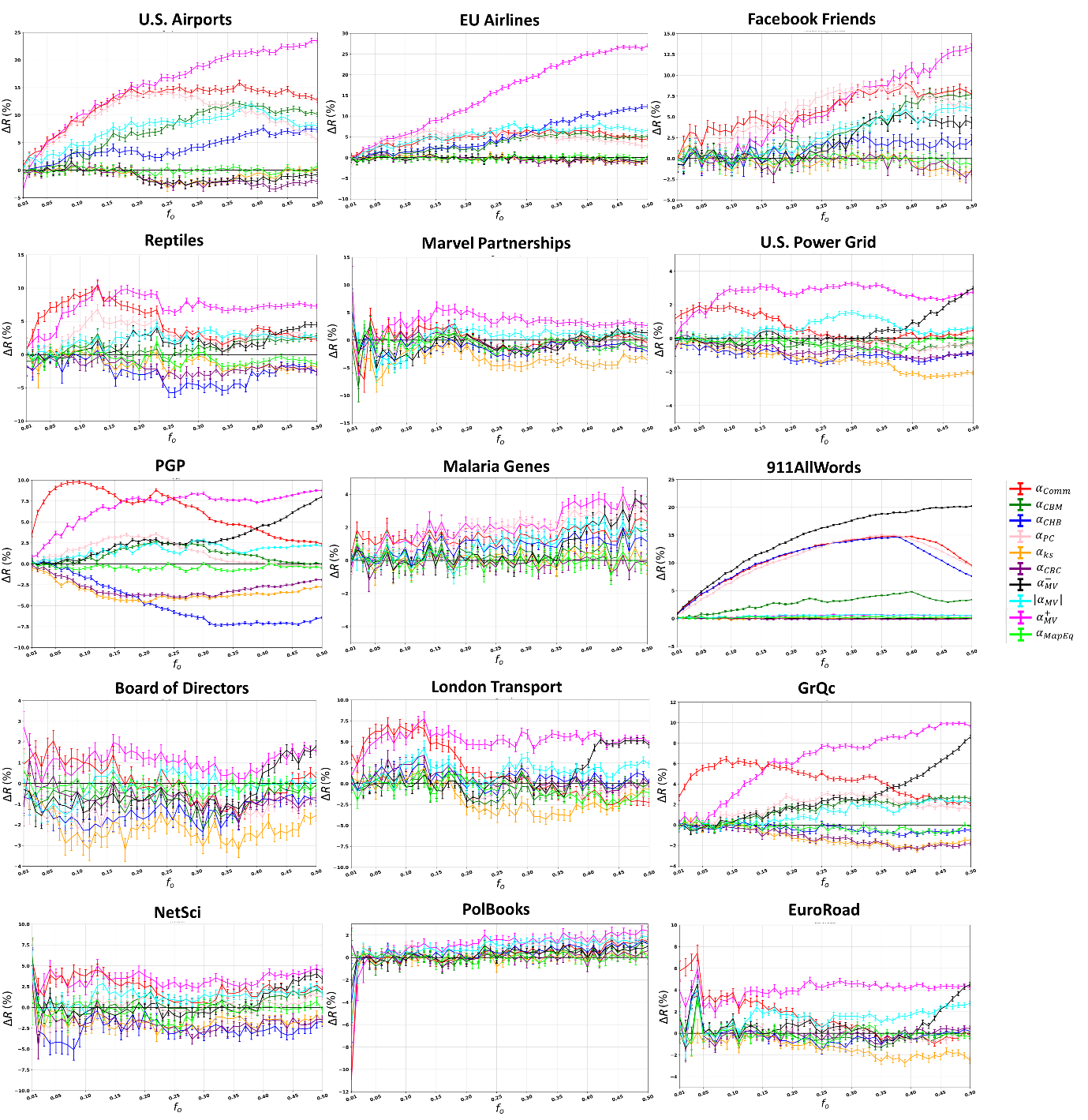


Figure 4 - The trends of the SIR model in fifteen real-world networks. The x-axis represents the budget availability or the fraction of initially infected nodes ($f_{o}$) and the y-axis represents the relative outbreak size ($\Delta R$).


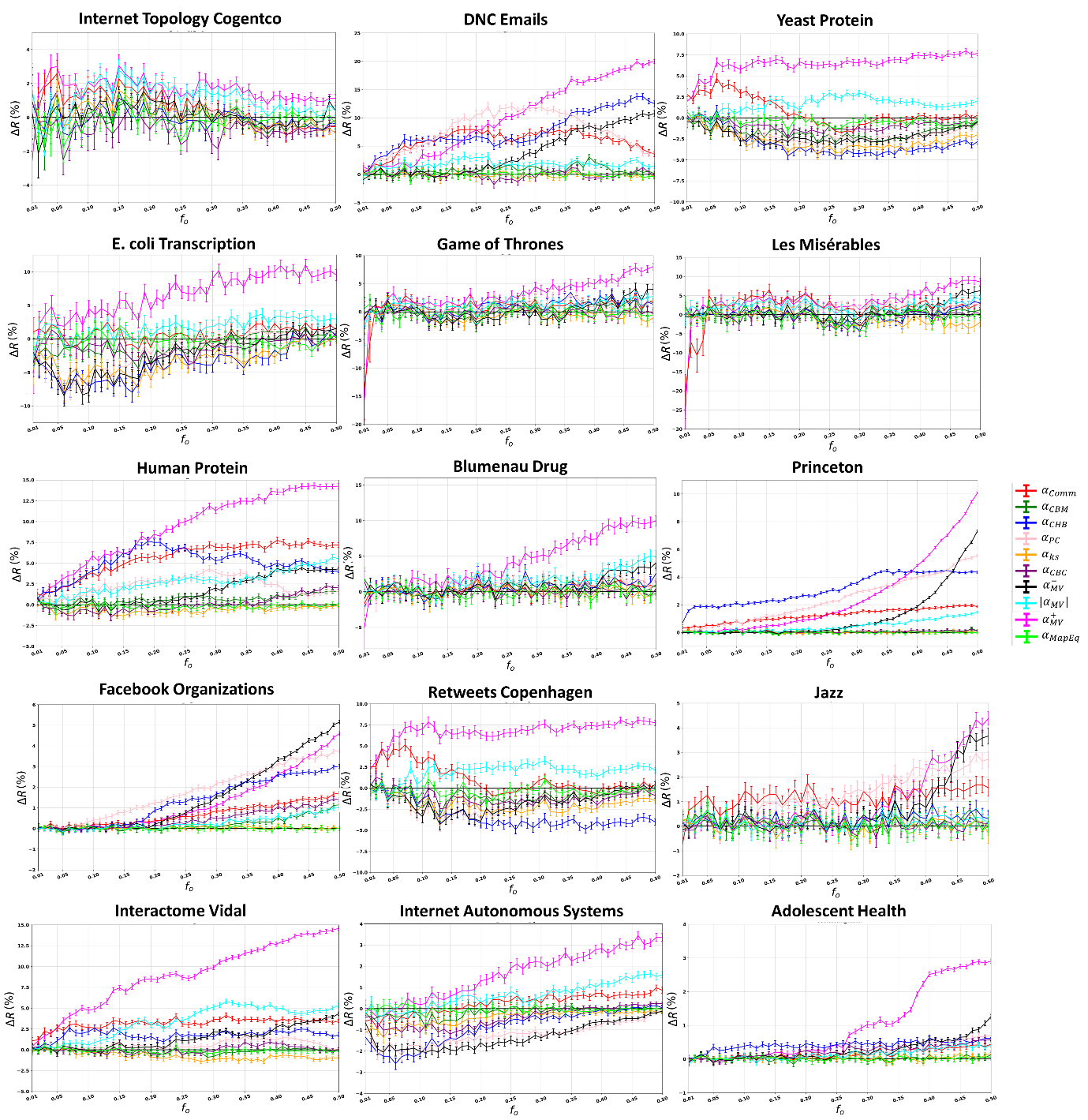


Figure 5 - The trends of the SIR model in fifteen real-world networks. The x-axis represents the budget availability or the fraction of initially infected nodes ($f_{o}$) and the y-axis represents the relative outbreak size ($\Delta R$).


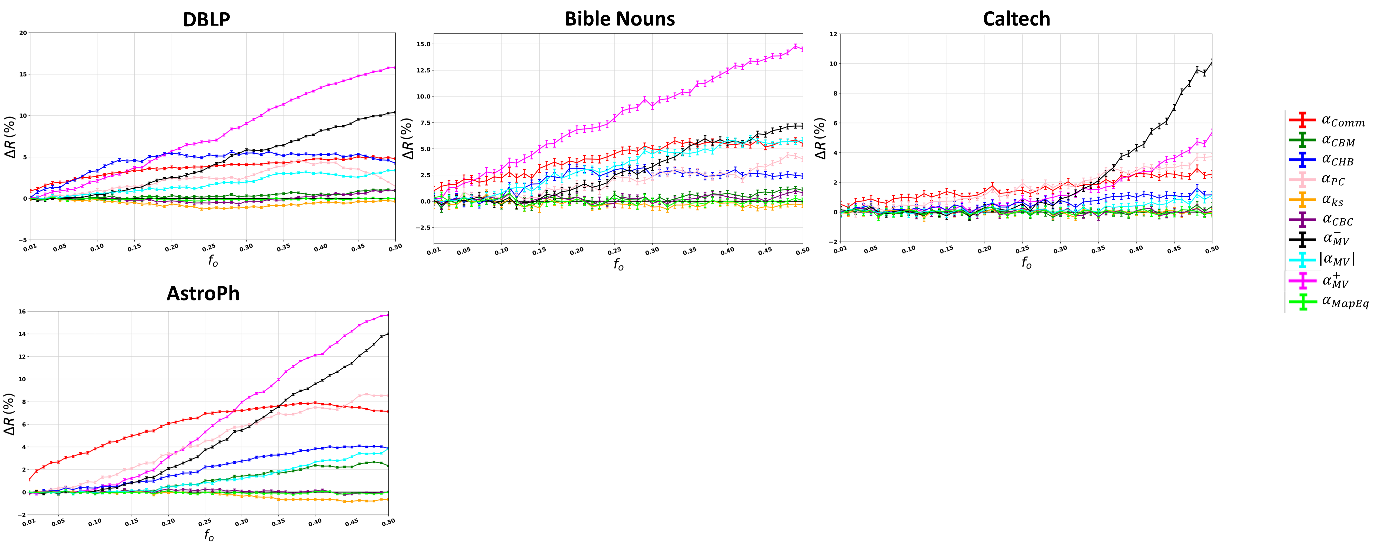


Figure 6 - The trends of the SIR model in four real-world networks. The x-axis represents the budget availability or the fraction of initially infected nodes ($f_{o}$) and the y-axis represents the relative outbreak size ($\Delta R$).

## Linear Threshold (LT) model


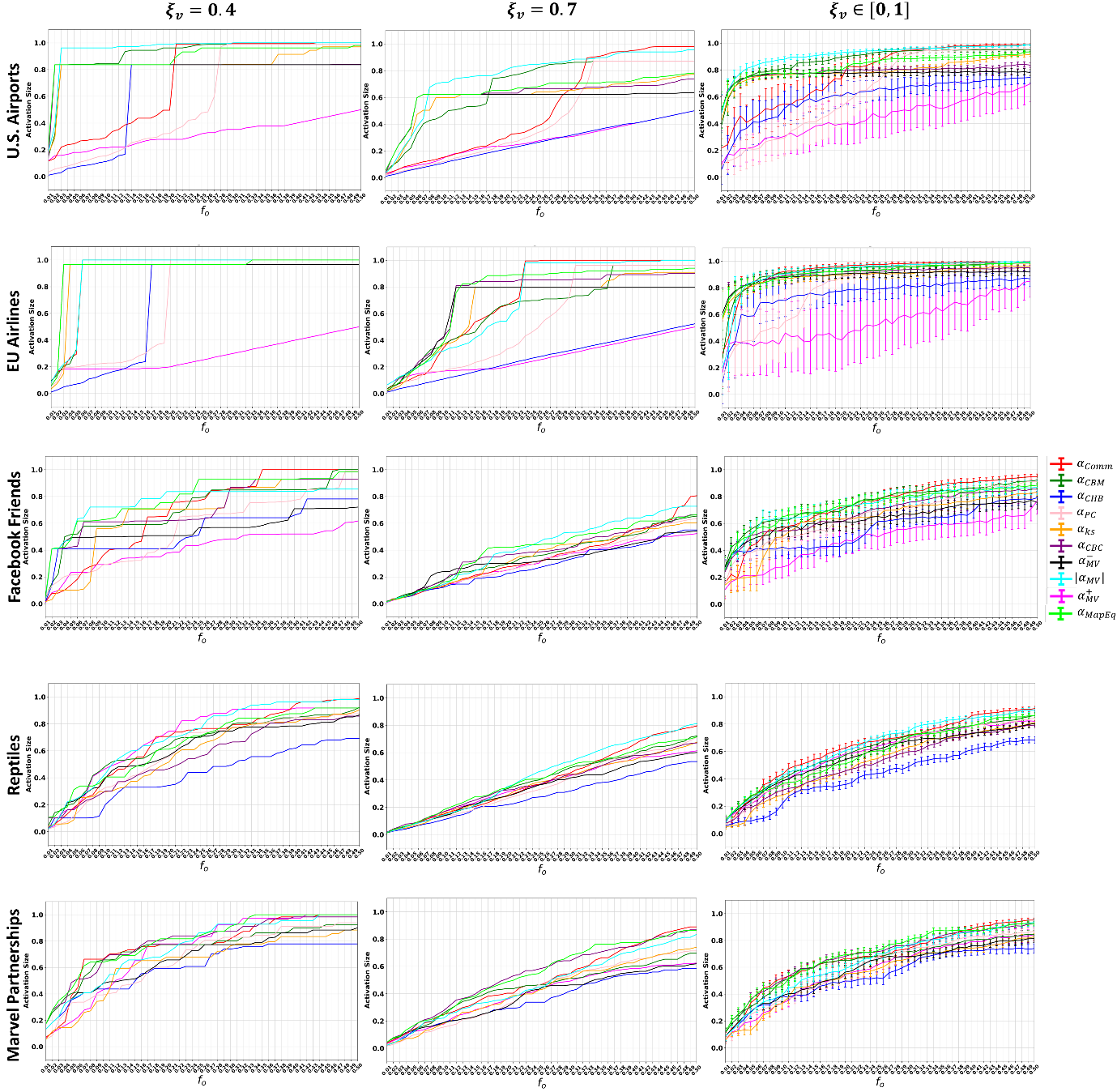


Figure 7 - The LT diffusion dynamics in six real-world networks with low, high, and random thresholds set on nodes ($\xi_{v}$). The x-axis represents the budget availability or the fraction of initially activated nodes ($f_{o}$) and the y-axis represents the activation rate ($A_{r}$).


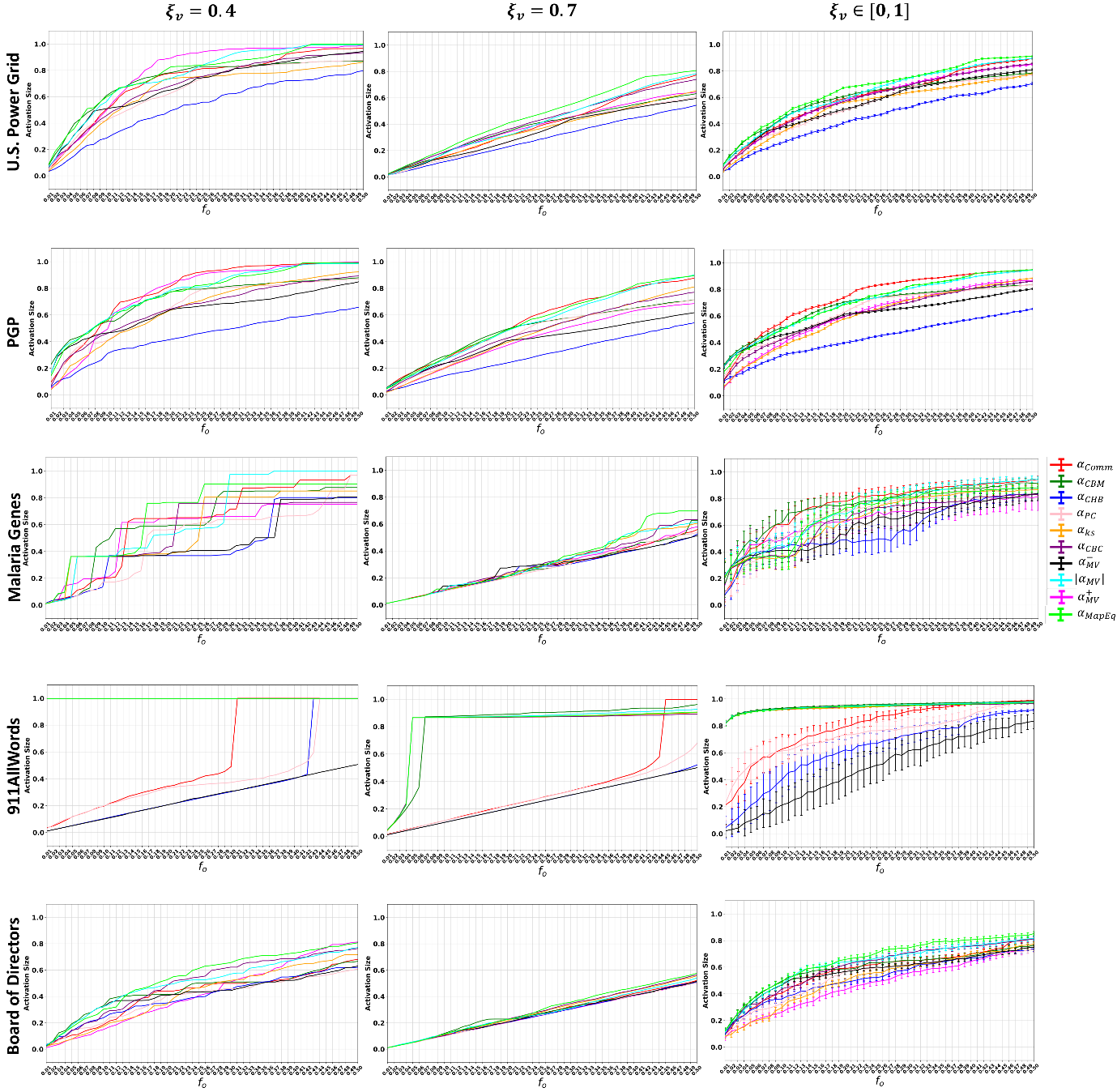


Figure 8 - The LT diffusion dynamics in six real-world networks with low, high, and random thresholds set on nodes ($\xi_{v}$). The x-axis represents the budget availability or the fraction of initially activated nodes ($f_{o}$) and the y-axis represents the activation rate ($A_{r}$).


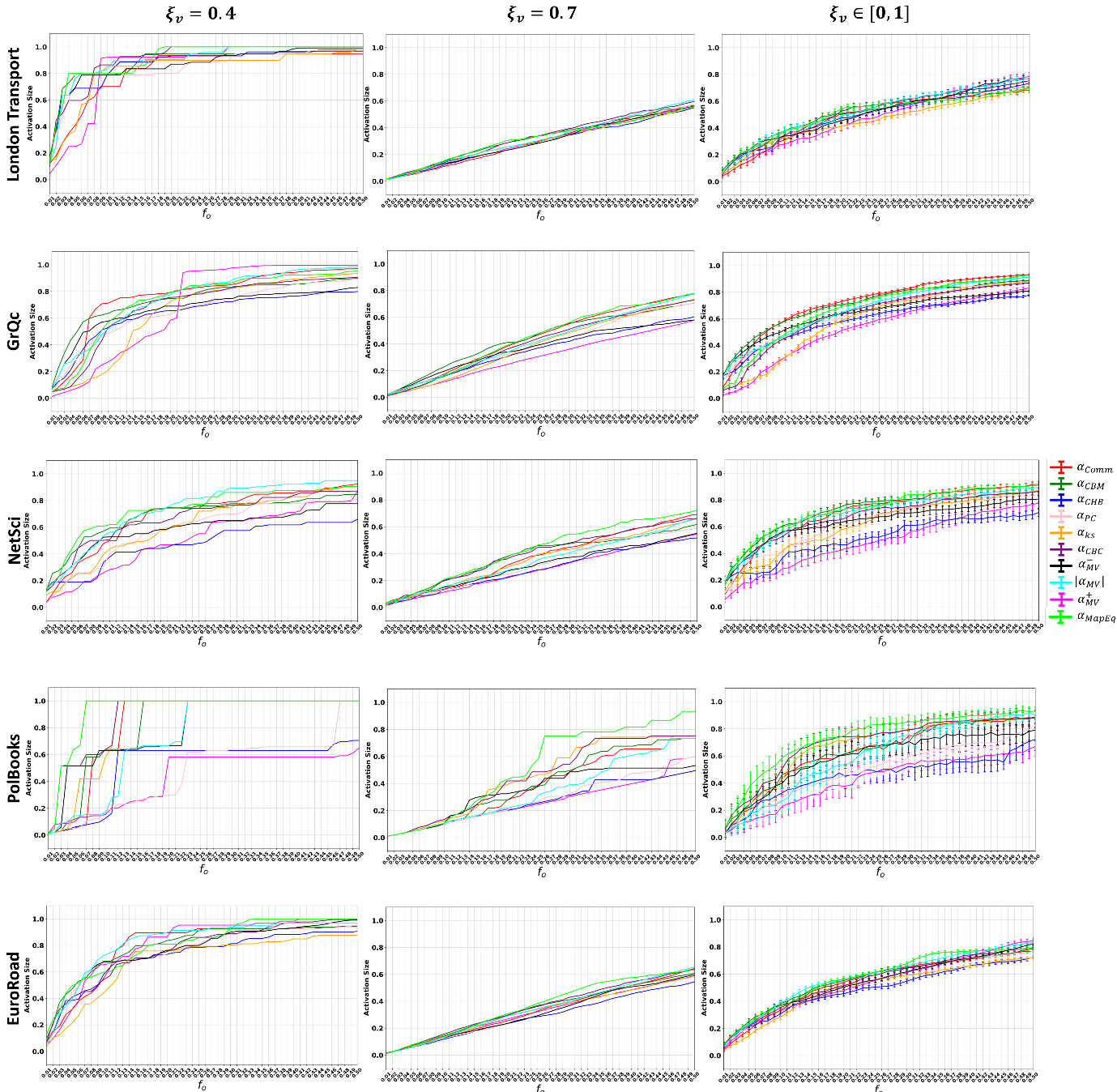


Figure 9 - The LT diffusion dynamics in six real-world networks with low, high, and random thresholds set on nodes ($\xi_{v}$). The x-axis represents the budget availability or the fraction of initially activated nodes ($f_{o}$) and the y-axis represents the activation rate ($A_{r}$).


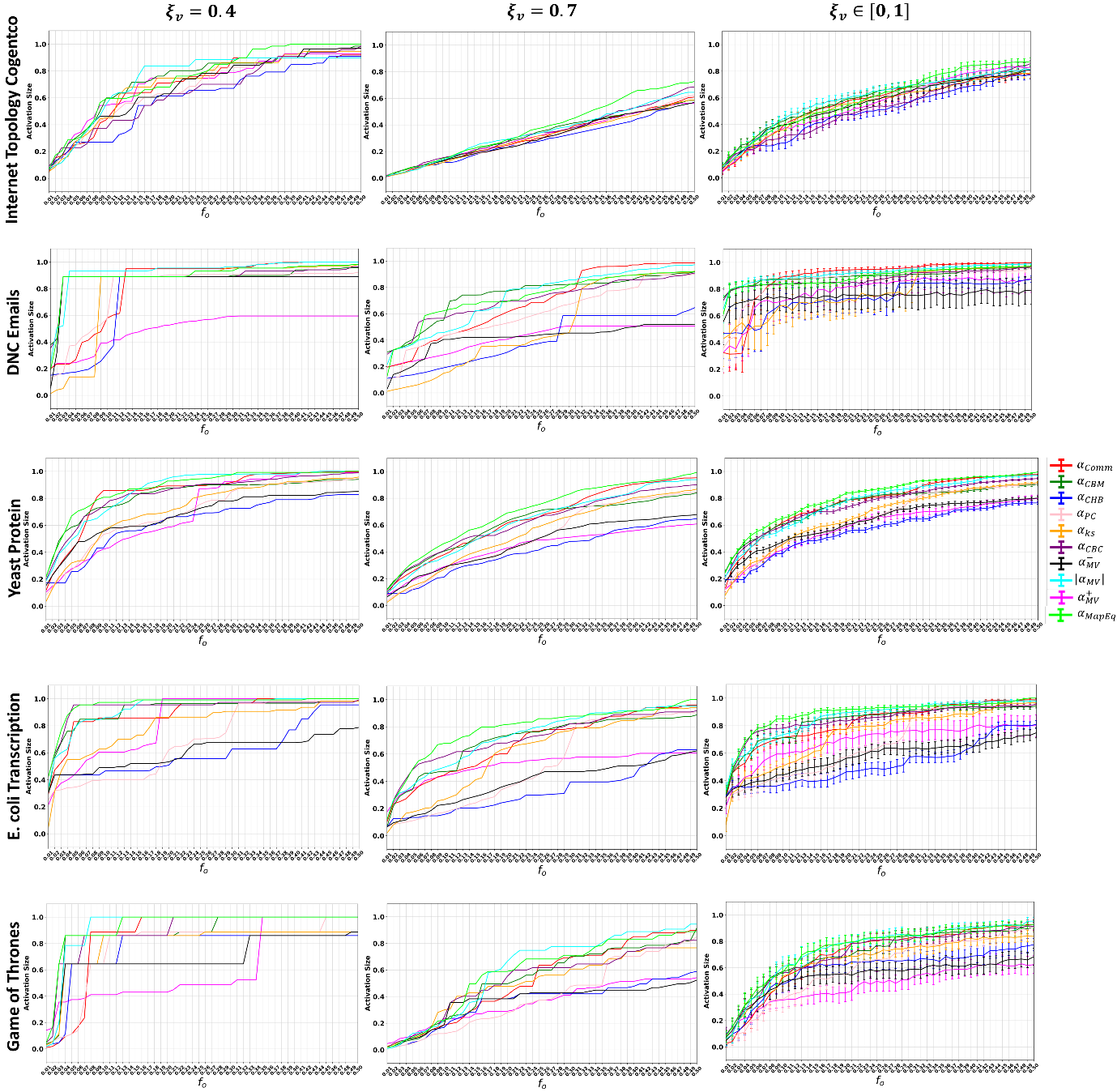


Figure 10 - The LT diffusion dynamics in six real-world networks with low, high, and random thresholds set on nodes ($\xi_{v}$). The x-axis represents the budget availability or the fraction of initially activated nodes ($f_{o}$) and the y-axis represents the activation rate ($A_{r}$).


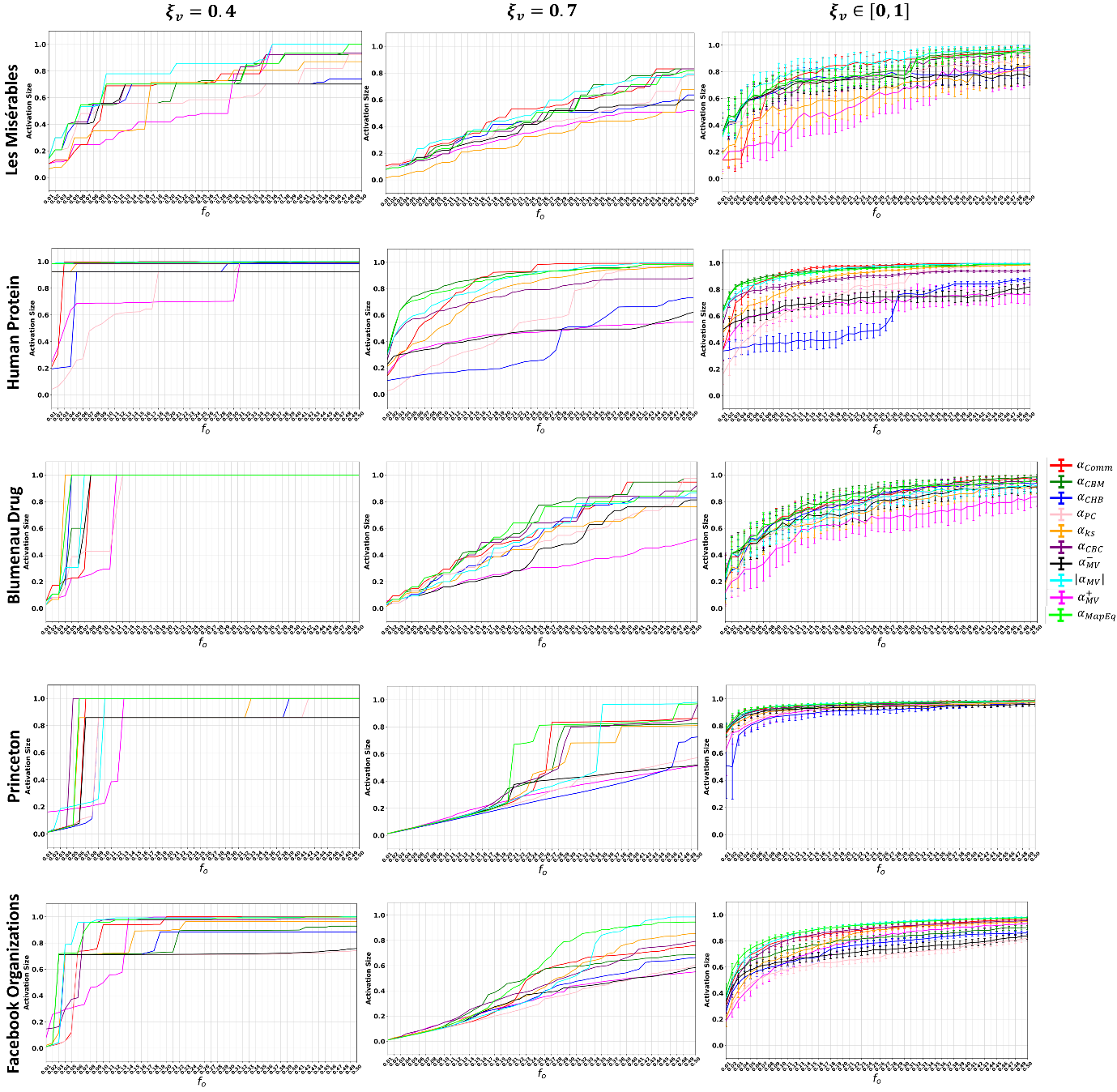


Figure 11 - The LT diffusion dynamics in six real-world networks with low, high, and random thresholds set on nodes ($\xi_{v}$). The x-axis represents the budget availability or the fraction of initially activated nodes ($f_{o}$) and the y-axis represents the activation rate ($A_{r}$).


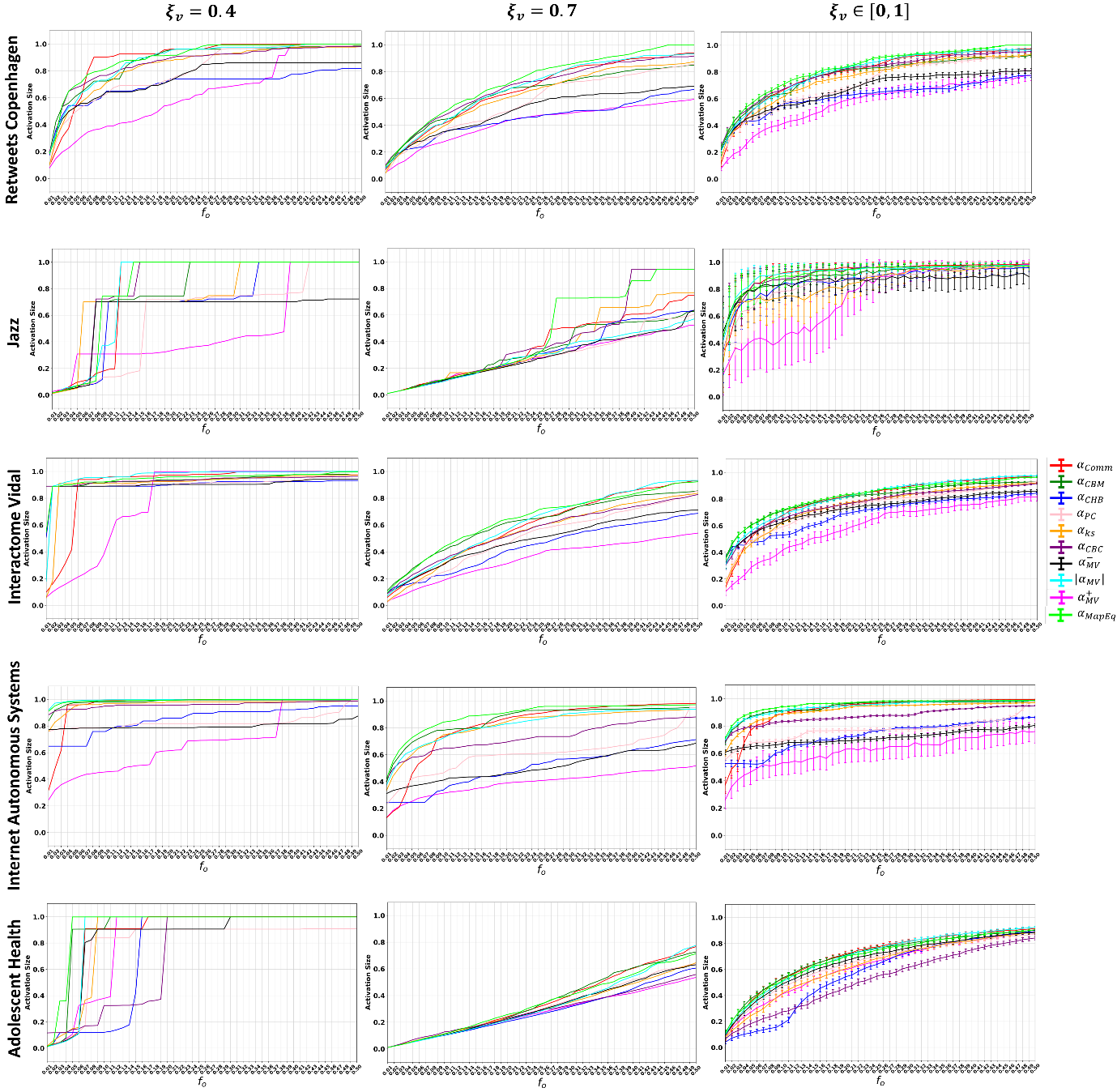


Figure 12 - The LT diffusion dynamics in six real-world networks with low, high, and random thresholds set on nodes ($\xi_{v}$). The x-axis represents the budget availability or the fraction of initially activated nodes ($f_{o}$) and the y-axis represents the activation rate ($A_{r}$).


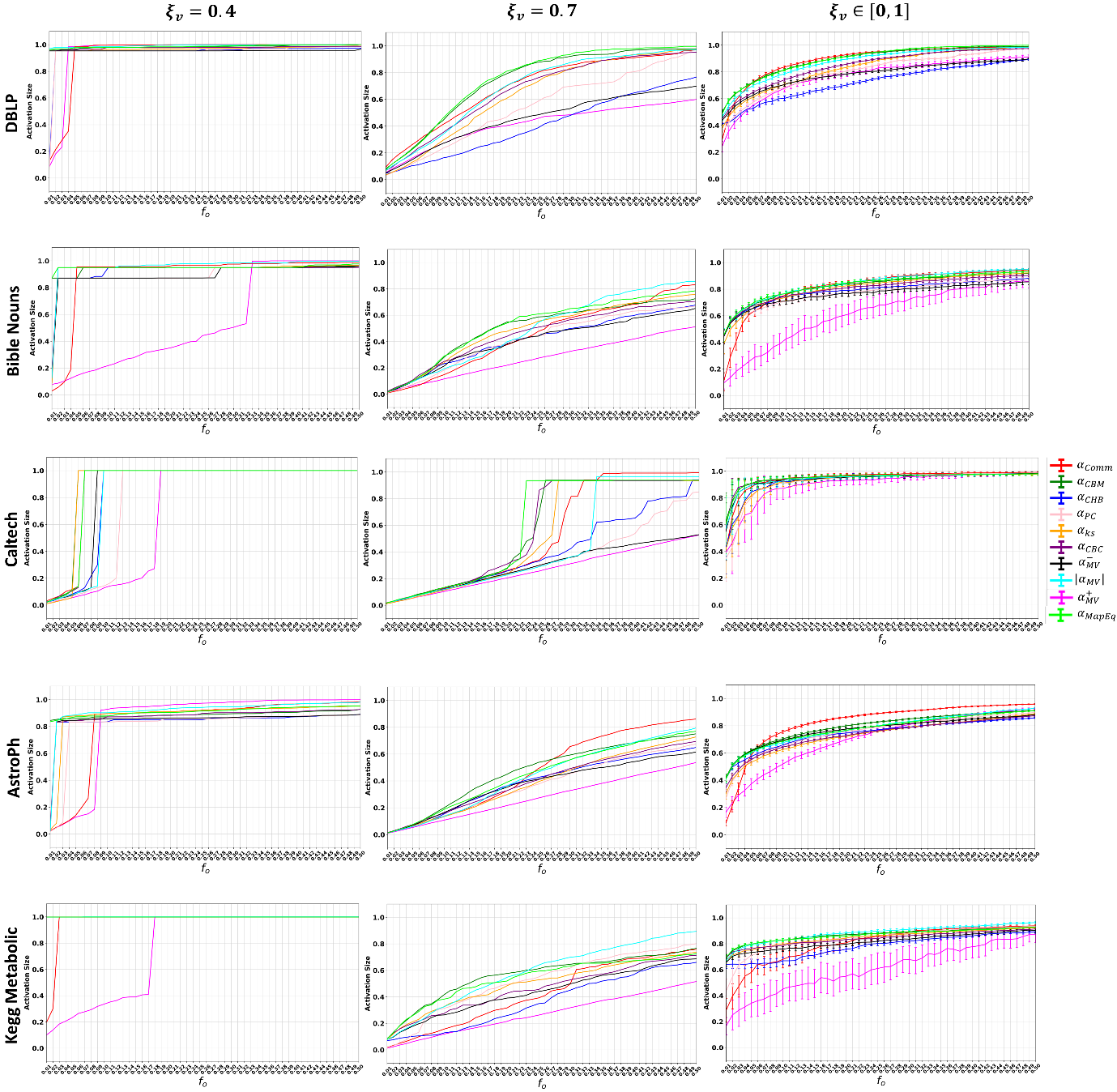


Figure 13 - The LT diffusion dynamics in six real-world networks with low, high, and random thresholds set on nodes ($\xi_{v}$). The x-axis represents the budget availability or the fraction of initially activated nodes ($f_{o}$) and the y-axis represents the activation rate ($A_{r}$).


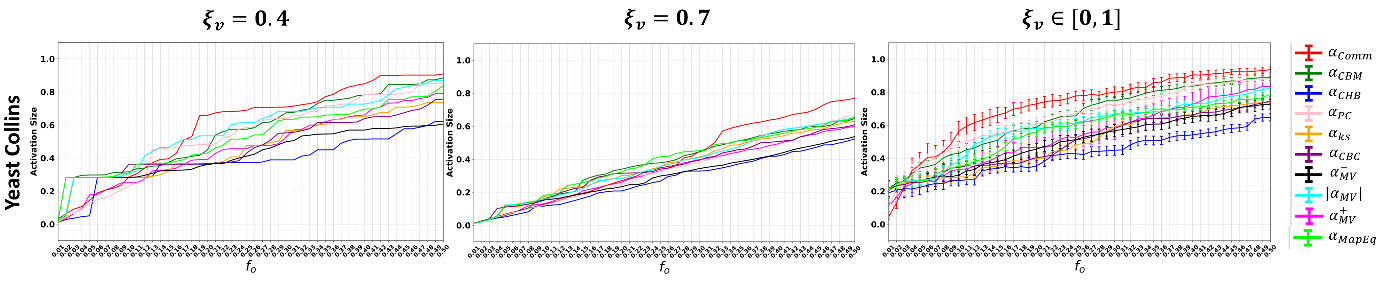


Figure 14 - The LT diffusion dynamics in one real-world network with low, high, and random thresholds set on nodes ($\xi_{v}$). The x-axis represents the budget availability or the fraction of initially activated nodes ($f_{o}$) and the y-axis represents the activation rate ($A_{r}$).

## Independent Cascade (IC) model


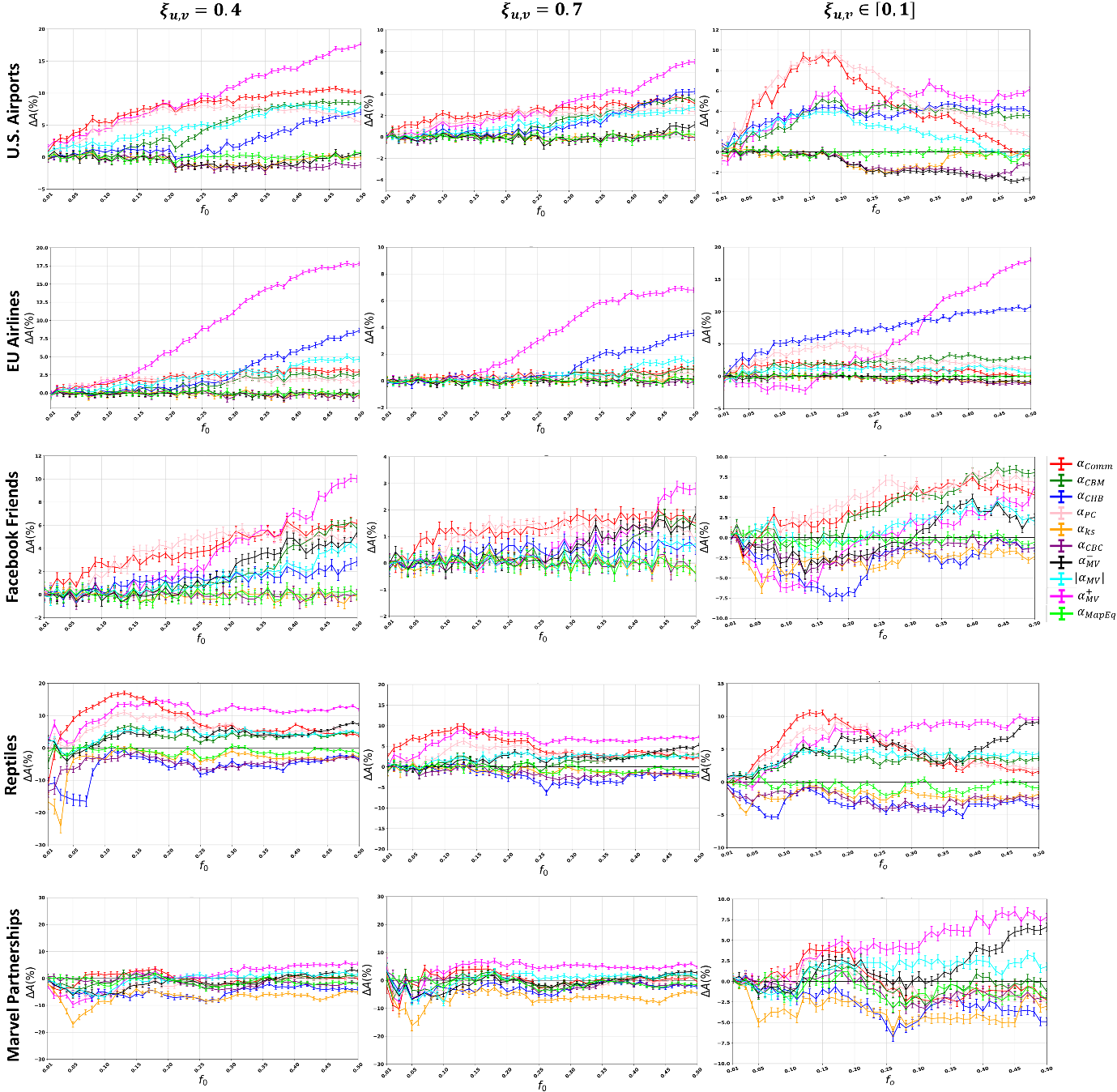


Figure 15 – The IC diffusion dynamics in six real-world networks with low, high, and random thresholds set on edges ($\xi_{u,v}$). The x-axis represents the budget availability or the fraction of initially activated nodes ($f_{o}$) and the y-axis represents the relative activation size ($\Delta A$).


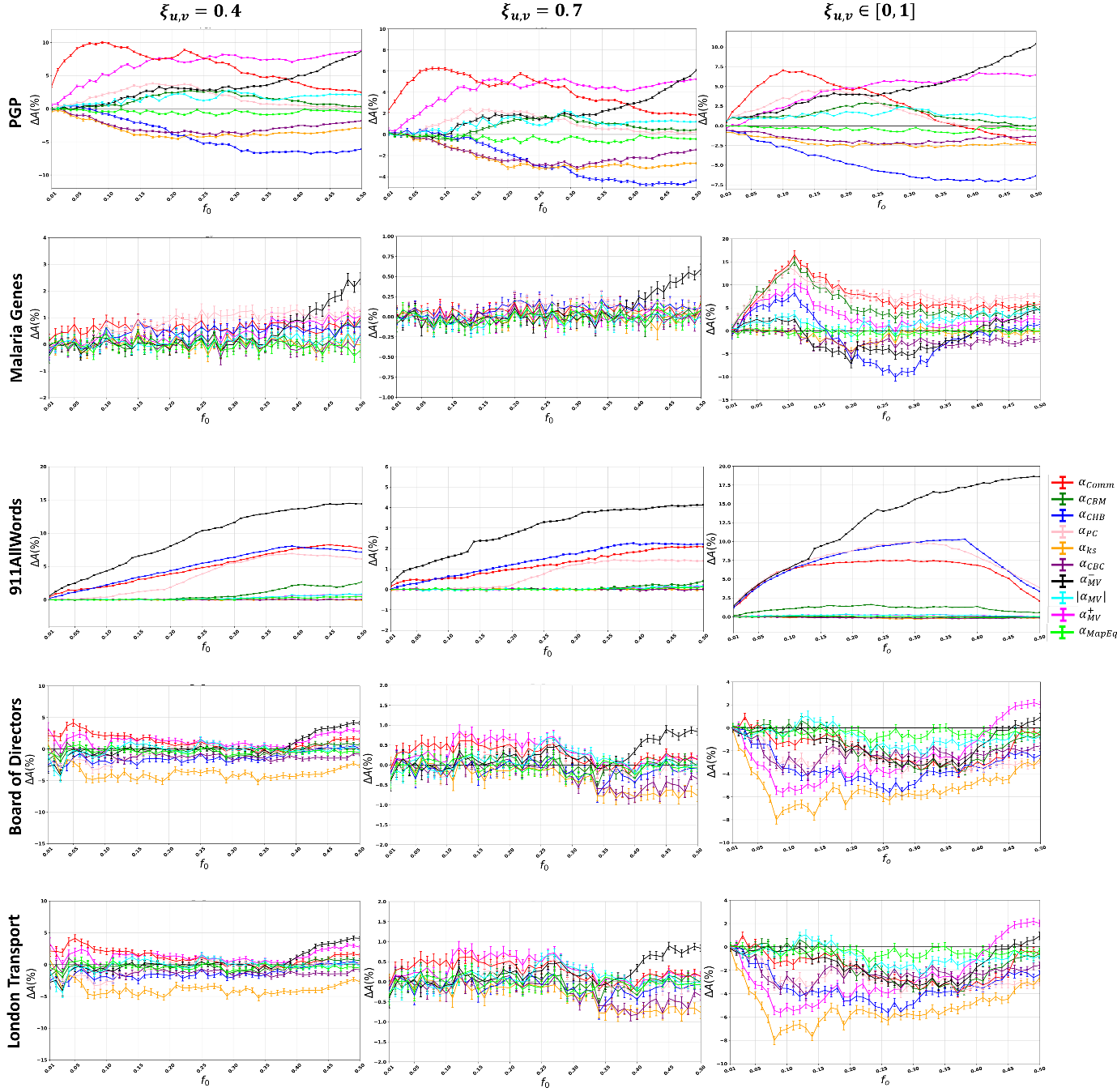


Figure 16 - The IC diffusion dynamics in six real-world networks with low, high, and random thresholds set on edges ($\xi_{u,v}$). The x-axis represents the budget availability or the fraction of initially activated nodes ($f_{o}$) and the y-axis represents the relative activation size ($\Delta A$).


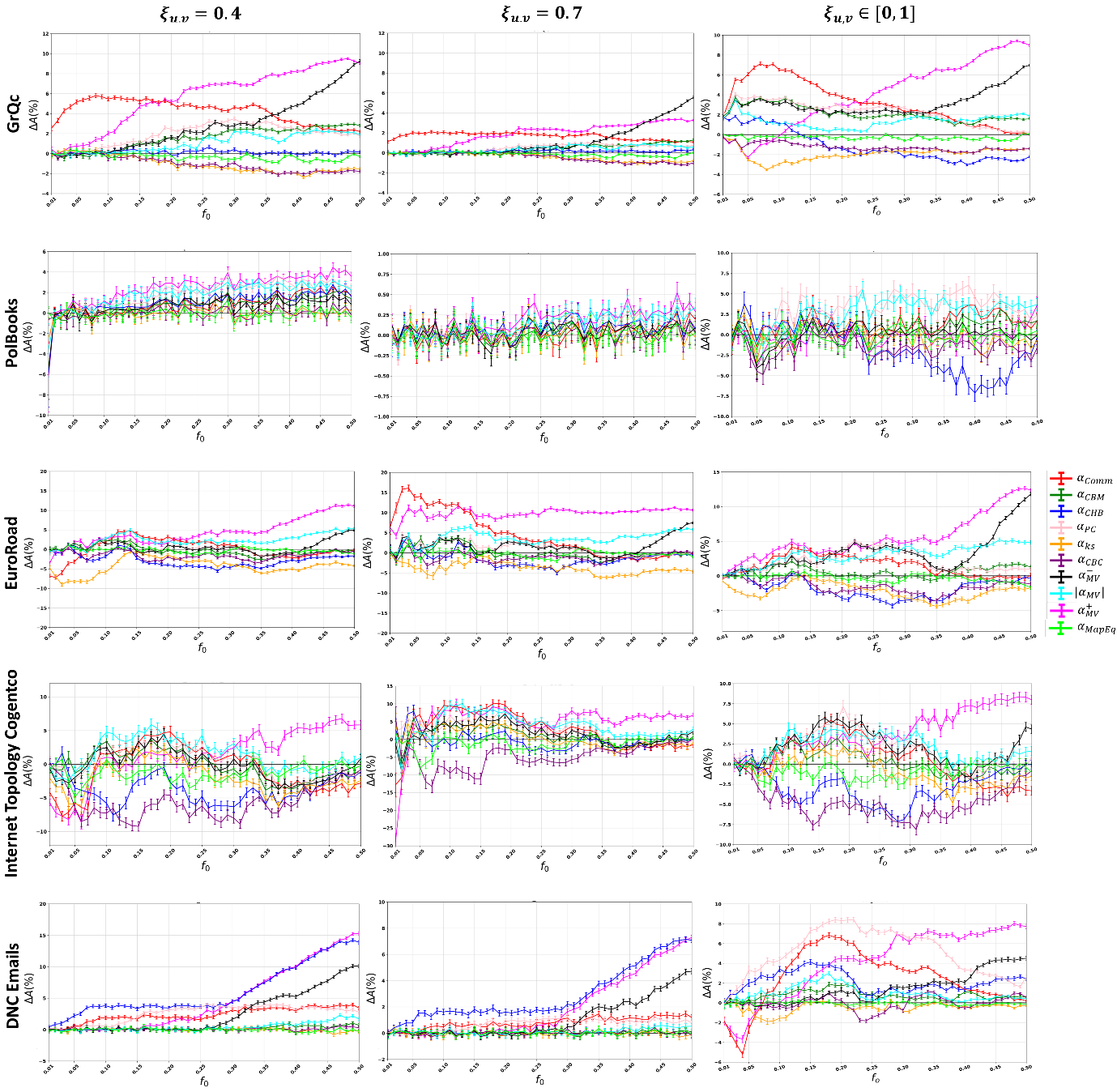


Figure 17 - The IC diffusion dynamics in six real-world networks with low, high, and random thresholds set on edges ($\xi_{u,v}$). The x-axis represents the budget availability or the fraction of initially activated nodes ($f_{o}$) and the y-axis represents the relative activation size ($\Delta A$).


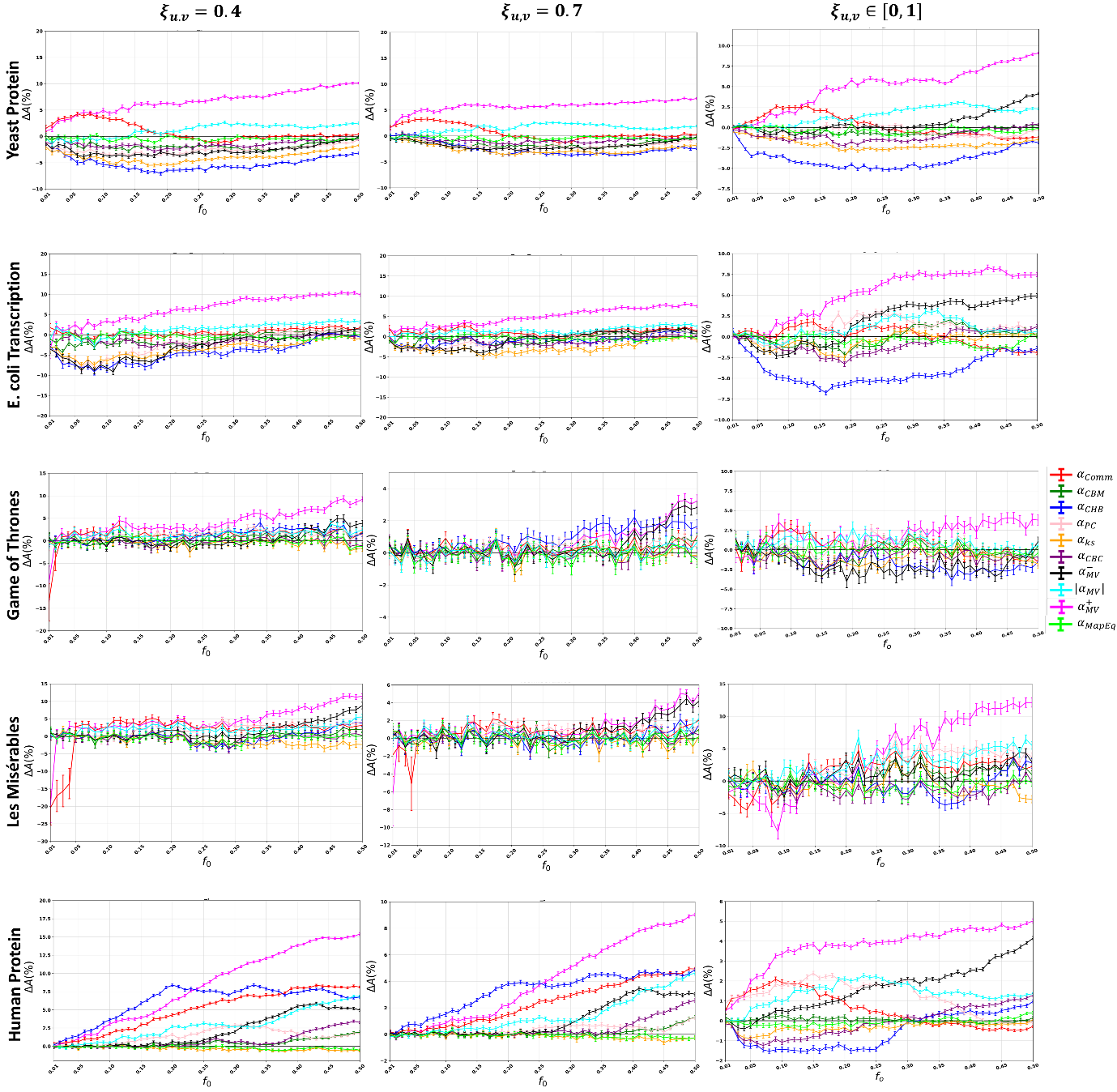


Figure 18 - The IC diffusion dynamics in six real-world networks with low, high, and random thresholds set on edges ($\xi_{u,v}$). The x-axis represents the budget availability or the fraction of initially activated nodes ($f_{o}$) and the y-axis represents the relative activation size ($\Delta A$).


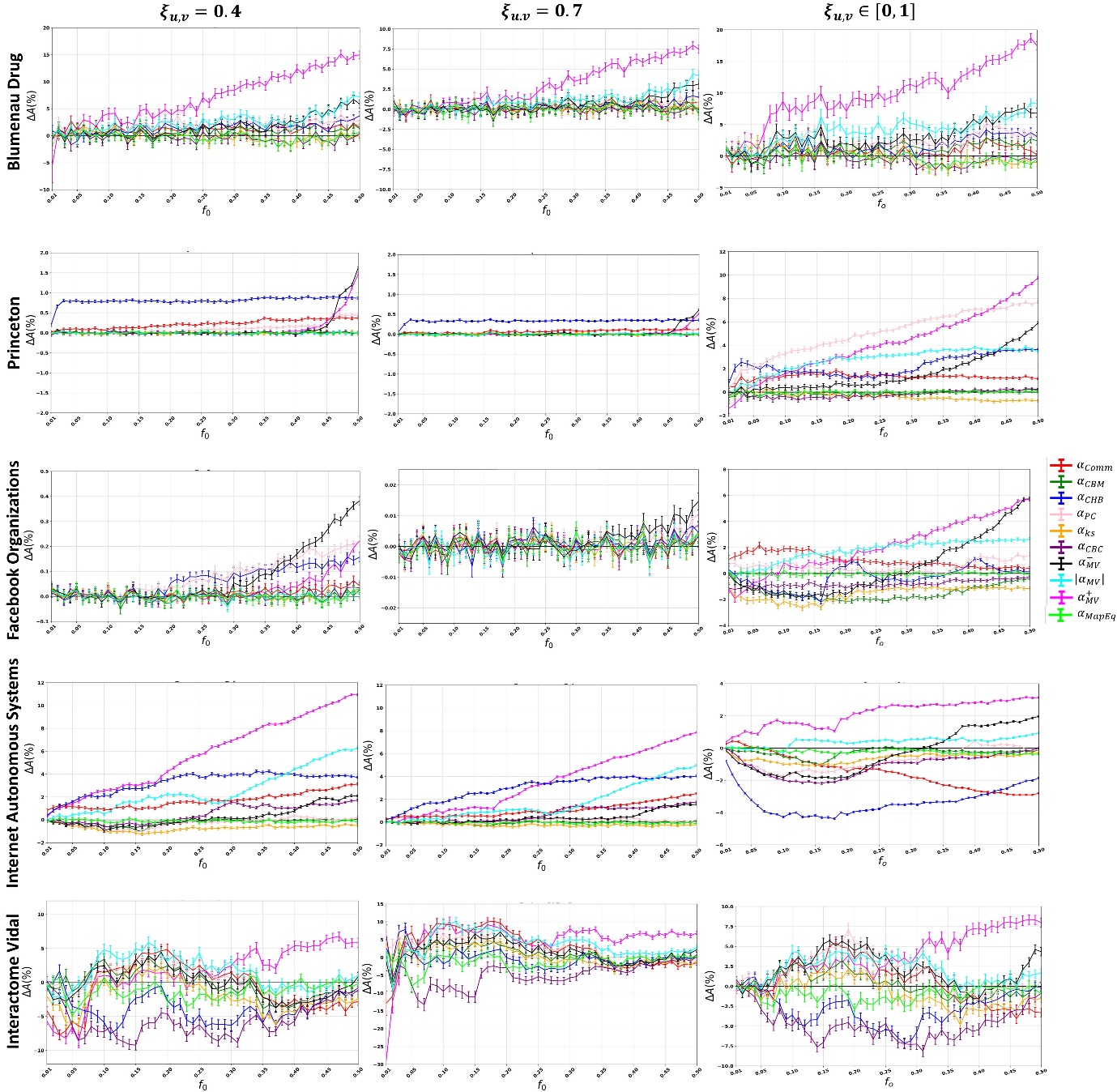


Figure 19 - The IC diffusion dynamics in six real-world networks with low, high, and random thresholds set on edges ($\xi_{u,v}$). The x-axis represents the budget availability or the fraction of initially activated nodes ($f_{o}$) and the y-axis represents the relative activation size ($\Delta A$).


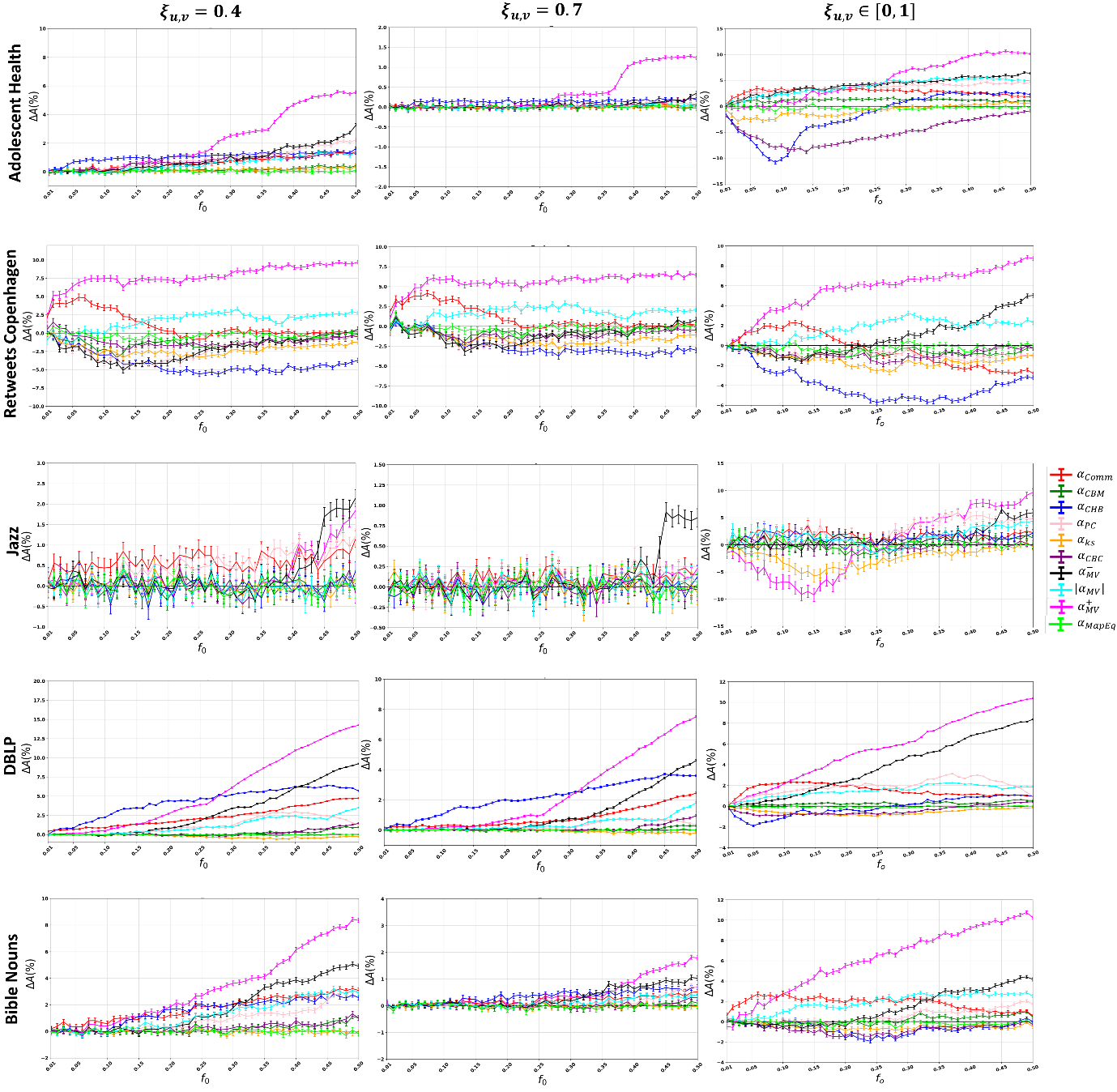


Figure 20 - The IC diffusion dynamics in six real-world networks with low, high, and random thresholds set on edges ($\xi_{u,v}$). The x-axis represents the budget availability or the fraction of initially activated nodes ($f_{o}$) and the y-axis represents the relative activation size ($\Delta A$).


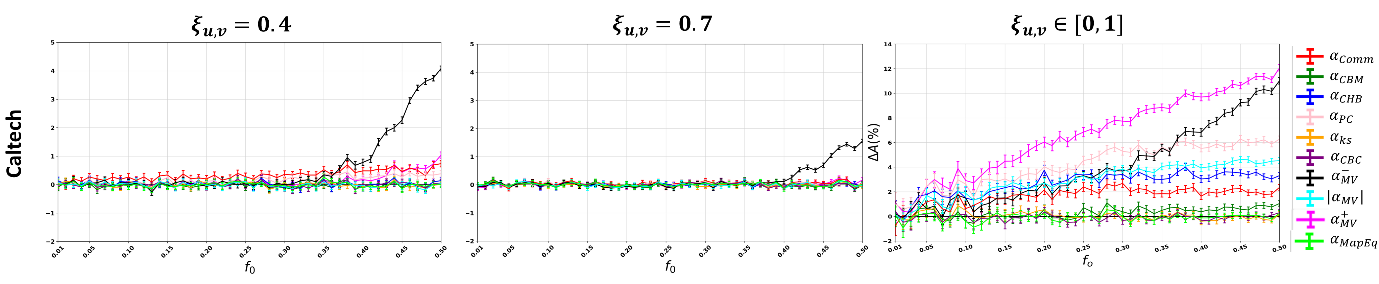


Figure 21 - The IC diffusion dynamics in one real-world network with low, high, and random thresholds set on edges ($\xi_{u,v}$). The x-axis represents the budget availability or the fraction of initially activated nodes ($f_{o}$) and the y-axis represents the relative activation size ($\Delta A$).

**Supplementary References**

[1] Guimera, R., & Nunes Amaral, L. A. (2005). Functional cartography of complex metabolic networks. *nature*, *433*(7028), 895-900.

[2] Zhao, Z., Wang, X., Zhang, W., & Zhu, Z. (2015). A community-based approach to identifying influential spreaders. *Entropy*, *17*(4), 2228-2252.

[3] Gupta, N., Singh, A., & Cherifi, H. (2016). Centrality measures for networks with community structure. *Physica A: Statistical Mechanics and its Applications*, *452*, 46-59.

[4] Luo, S. L., Gong, K., & Kang, L. (2016). Identifying influential spreaders of epidemics on community networks. *arXiv preprint arXiv:1601.07700*.

[5] Tulu, M. M., Hou, R., & Younas, T. (2018). Identifying influential nodes based on community structure to speed up the dissemination of information in complex network. *IEEE access*, *6*, 7390-7401.

[6] Ghalmane, Z., Hassouni, M. E., & Cherifi, H. (2019). Immunization of networks with non-overlapping community structure. *Social Network Analysis and Mining*, *9*, 1-22.

[7] Magelinski, T., Bartulovic, M., & Carley, K. M. (2021). Measuring node contribution to community structure with modularity vitality. *IEEE Transactions on Network Science and Engineering*, *8*(1), 707-723.

[8] Blöcker, C., Nieves, J. C., & Rosvall, M. (2022). Map equation centrality: community-aware centrality based on the map equation. *Applied Network Science*, *7*(1), 56.

[9] Lancichinetti, A., Fortunato, S., & Radicchi, F. (2008). Benchmark graphs for testing community detection algorithms. *Physical review E*, 78(4), 046110.

[10] Wang, Wei, et al. "Predicting the epidemic threshold of the susceptible-infected- recovered model." *Scientific reports* 6.1 (2016): 1-12.

[11] Rosvall, M. & Bergstrom, C. T. Maps of random walks on complex networks reveal community structure. *Proc. Natl. Acad. Sci*. 105, 1118–1123 (2008)

[12] Blondel, V. D., Guillaume, J.-L., Lambiotte, R. & Lefebvre, E. Fast unfolding of communities in large networks. J*. statistical mechanics: theory experiment* 2008, P10008 (2008).

[13] Peixoto, T. P. "The netzschleuder network catalogue and repository (2020)." URL https://networks. skewed. de (2020).

[14] Rossi, R., & Ahmed, N. (2015, March). The network data repository with interactive graph analytics and visualization. *In Twenty-ninth AAAI conference on artificial intelligence*.

[15] Kunegis, Jérôme. "Handbook of Network Analysis [KONECT--the Koblenz Network Collection]." *arXiv preprint* arXiv:1402.5500 (2014).

[16] Clauset, Aaron, Ellen Tucker, and Matthias Sainz. "The Colorado index of complex networks." Retrieved July 20.2018 (2016): 22.

[17] Rozemberczki, B., & Sarkar, R. (2020, October). Characteristic functions on graphs: Birds of a feather, from statistical descriptors to parametric models. *In Proceedings of the 29th ACM international conference on information & knowledge management* (pp. 1325-1334).

[18] Latora, V., Nicosia, V., & Russo, G. (2017). Complex networks: principles, methods and applications. Cambridge University Press.

1. https://ndlib.readthedocs.io/ [↑](#footnote-ref-1)
